# Isolation and Characterization of Anti-Adenoviral Secondary Metabolites from Marine Actinobacteria

**DOI:** 10.3390/md12020799

**Published:** 2014-01-28

**Authors:** Mårten Strand, Marcus Carlsson, Hanna Uvell, Koushikul Islam, Karin Edlund, Inger Cullman, Björn Altermark, Ya-Fang Mei, Mikael Elofsson, Nils-Peder Willassen, Göran Wadell, Fredrik Almqvist

**Affiliations:** 1 Division of Virology, Department of Clinical Microbiology, Umeå University, Umeå SE-90185, Sweden; E-Mails: marten.strand@climi.umu.se (M.S.); koushikul.islam@climi.umu.se (K.I.); karin.edlund@climi.umu.se (K.E.); mei.ya-fang@climi.umu.se (Y.-F.M.); goran.wadell@climi.umu.se (G.W.); 2 Laboratories for Chemical Biology, Department of Chemistry, Umeå University, Umeå SE-90187, Sweden; E-Mails: marcus.carlsson@chem.umu.se (M.C.); hanna.uvell@chem.umu.se (H.U.); inger.cullman@chem.umu.se (I.C.); mikael.elofsson@chem.umu.se (M.E.); 3 Department of Chemistry, Faculty of Science and Technology, University of Tromsø, Tromsø 9037, Norway; E-Mails: bjorn.altermark@uit.no (B.A.); nils-peder.willassen@uit.no (N.-P.W.)

**Keywords:** adenovirus, antiviral, natural products, secondary metabolites, marine actinobacteria, extract screening, butenolides

## Abstract

Adenovirus infections in immunocompromised patients are associated with high mortality rates. Currently, there are no effective anti-adenoviral therapies available. It is well known that actinobacteria can produce secondary metabolites that are attractive in drug discovery due to their structural diversity and their evolved interaction with biomolecules. Here, we have established an extract library derived from actinobacteria isolated from Vestfjorden, Norway, and performed a screening campaign to discover anti-adenoviral compounds. One extract with anti-adenoviral activity was found to contain a diastereomeric 1:1 mixture of the butenolide secondary alcohols **1a** and **1b**. By further cultivation and analysis, we could isolate **1a** and **1b** in different diastereomeric ratio. In addition, three more anti-adenoviral butenolides **2**, **3** and **4** with differences in their side-chains were isolated. In this study, the anti-adenoviral activity of these compounds was characterized and substantial differences in the cytotoxic potential between the butenolide analogs were observed. The most potent butenolide analog **3** displayed an EC_50_ value of 91 μM and no prominent cytotoxicity at 2 mM. Furthermore, we propose a biosynthetic pathway for these compounds based on their relative time of appearance and structure.

## 1. Introduction

Secondary metabolites produced by actinobacteria have been studied extensively since the 1950s and have been the most economically and biotechnologically valuable source for discovery of new compounds. The characterization of secondary metabolites from actinobacteria has yielded many important drug leads, later developed into anti-microbial (vancomycin, chloramphenicol and tetracycline) anti-cancer (daunorubicin and bleomycin) and immunosuppressive agents (rapamycin). However, the discovery rate of new drug candidates from terrestrial sources has decreased notably, whereas the re-isolation of known compounds has increased [1,2]. The emerging threat of drug-resistant microbes and the globally increasing cancer rates makes it crucial to produce novel and unique drugs, possibly from new sources of actinobacteria. Likewise, identification of new biological applications for known compounds is equally desirable in drug discovery.

The greatest biodiversity is found in the oceans that cover more than 70% of the Earth’s surface [3]. The marine microbial diversity is largely unexplored but has recently gained much attention as a new source for discovery of new drug candidates [4,5]. In particular, the characterization and the biological evaluation of the secondary metabolites from marine actinobacteria are to a large extent still unexploited and of great interest for research and drug development.

The scope of this paper is to find molecules with anti-adenoviral activity from actinobacteria isolated in the Arctic sea. Today, there is an urgent need for development of new therapeutics for treatment of adenovirus infections. Human adenovirus (HAdV) consists of 57 types divided into seven species (A–G) which can cause disease in the respiratory, intestinal, and urinary tracts as well as in the eyes and liver [6]. These infections are often mild and subclinical in otherwise healthy individuals but in patients with impaired or suppressed immune system, adenovirus infections are associated with disseminated infections with high mortality rates. In hematopoietic bone marrow transplant recipients, a disseminated adenovirus infection is associated with mortality rates up to 60% and even higher rates are found in pediatric patients [7,8]. Currently, there are no approved antiviral therapies for the treatment of adenovirus, although the need is great.

**Figure 1:**
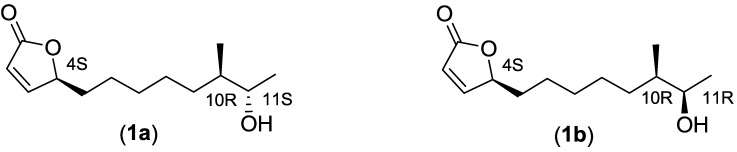
Structures of the diastereomeric mixture of the butenolides **1a** and **1b** that was responsible for the anti-adenoviral activity in the identified extract.

**Figure 2:**
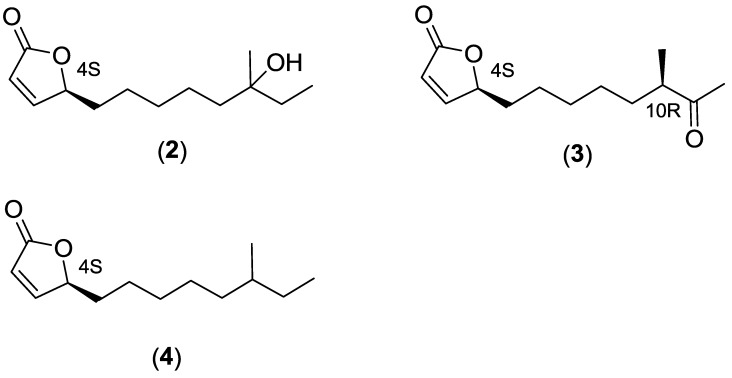
Structures of the isolated butenolides, **2**–**4**.

Screening of compound libraries is efficient for the identification of new bioactive compounds that can serve as starting points for drug discovery. We have previously developed a whole-cell based viral replication reporter gene assay utilizing a replication competent green fluorescent protein (GFP) expressing vector based on adenovirus type 11 (RCAd11GFP) [9,10]. This assay was used to screen novel synthetic small molecule inhibitors of viral replication [10] that have been further explored by chemical optimization and evaluation [11,12]. In this paper, we expand our screening-based approach and describe the screening of ethyl acetate (EtOAc) extracts derived from actinobacteria originating from Vestfjorden, Norway, and subsequent isolation and identification of anti-adenoviral butenolides **1**–**4** (Figure 1 and Figure 2) produced by a marine Streptomyces strain (*Streptomyces* sp. AW28M48). We propose a biosynthetic pathway for the butenolides **1**–**4** based on their relative time of appearance during cultivation and their structure.

## 2. Results and Discussion

### 2.1. Antiviral Screening

#### 2.1.1. Establishment of an EtOAc Extract Library Originating from Marine Actinobacteria

During a research expedition in Vestfjorden, Norway, actinobacteria were isolated from sediment and biota samples. A total of 57 actinobacteria were isolated. A crude EtOAc extract library was established by cultivating each bacterium in eight different media for five days at 26 °C. After cultivation, the bacteria were pelleted and the media were extracted with EtOAc under both acidic and basic conditions. A total number of 912 extracts were obtained and re-dissolved in DMSO. The extracts were put in microtiter plates suitable for screening.

#### 2.1.2. Screening for Anti-Adenoviral Activity in the EtOAc Extracts

**Figure 3:**
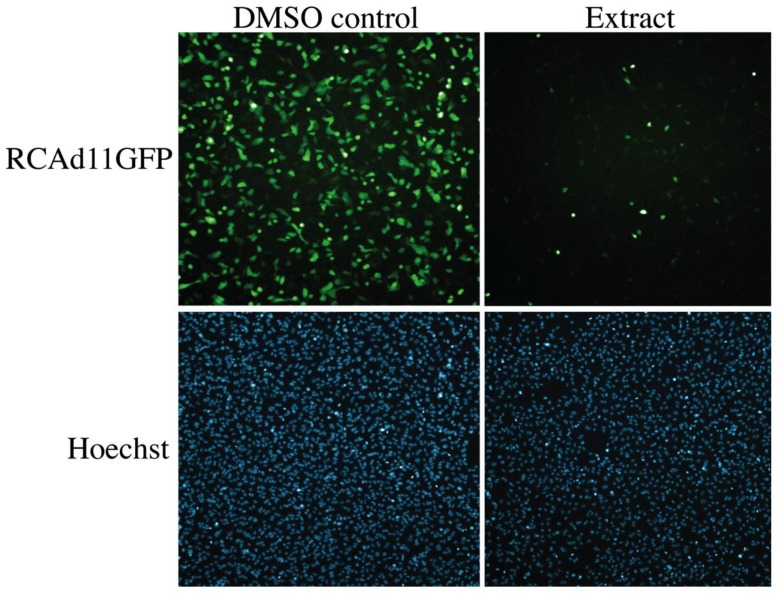
The observed anti-adenoviral activity of the actinobacteria EtOAc extract, dissolved in DMSO, that contained the diastereomeric mixture of the butenolide secondary alcohols **1a** and **1b** (Figure 1). The marine actinobacteria extract was diluted 1:200 and was added together with RCAd11GFP virus to A549 cells. Pictures were taken 24 h post infection at 50× magnification and display the viral GFP-expression (green) and nuclear staining with Hoechst (blue).

To assess the potential antiviral activity of the marine actinobacteria extracts a replication competent GFP-expressing adenoviral vector based on type 11 (RCAd11GFP) was used to infect A549 human lung adenocarcinoma epithelial cells, in the presence of extracts. The 912 extracts were evaluated 24 h post-infection by measuring the inhibition of the GFP-expression, as an indication of antiviral activity, and the potential morphological changes of the cellular nuclei that indicates cellular cytotoxicity, in a Thermo Fischer Scientific Cellomics^®^ ArrayScanVTi^®^. This facilitates the selection of bioactive extracts with consideration of both antiviral activity and low cytotoxicity. Among the 912 extracts, we found several extracts that displayed antiviral activity and no or moderate cytotoxicity on host A549 cells. Four extracts were further analyzed in depth using bioactivity-guided fractionation. The extract was fractionated using reverse phase HPLC and each fraction was tested for anti-adenoviral activity. In three of the extracts, we identified several compounds with previously known antiviral activity (data not shown). However, in the active fraction of the fourth extract, which originated from a Streptomyces strain (*Streptomyces* sp. AW28M48), we found a 1:1 diastereomeric mixture of the known butenolide secondary alcohols **1a** and **1b** (Figure 1) with previously reported antifouling activity and cytotoxicity [13,14,15]. These compounds have not previously been reported to have antiviral activity (Figure 3).

### 2.2. Isolation and Characterization of Butenolide Analogs

#### 2.2.1. Investigation of the Optimal Time of Cultivation for Production of the Butenolides **1a** and **1b**

In an attempt to increase the yield of the butenolide secondary alcohols **1a** and **1b**, an investigation of the cultivation time was performed using HPLC connected with a diode-array and evaporated light scattering detector. To get reproducible results, the same batch of frozen pre-culture was used for all inoculations. Two hundred milliliters media (PM2 with 4% sea salt) was inoculated with 2 mL pre-cultured *Streptomyces* sp. AW28M48, and cultured in 26 °C at 160 rpm shaking for two to ten days. Each day, a 5 mL sample was removed and extracted with EtOAc and analyzed with HPLC. In the original five day extract the butenolide secondary alcohols **1a** and **1b** were found in relatively low amounts as an inseparable 1:1 diastereomeric mixture. By shortening the time of cultivation to two days, one of the two diastereomers **1a** was isolated in 85% *de*. This coincides with the highest intensity for the peak corresponding to the butenolides **1a** and **1b** at the retention time of 9.04 min (Figure S1 in Supplementary Information). The intensity for the peak at 9.04 min decreased with longer cultivation times than two days; interestingly we could detect one peak at 9.55 min that showed inversed pattern, *i.e.*, increased after two days of cultivation. An additional small adjacent peak was also observed at 8.93 min, showing similar time of appearance as the butenolides **1a** and **1b**. The peaks at 9.55 min and 8.93 min were detected with similar UV-vis profiles (Figure S3 in Supplementary Information) to **1a** and **1b**, indicative of being analogs to the butenolide secondary alcohols. When the peak at 9.55 min was isolated, it was shown to be the oxidized butenolide ketone **3**, while the peak at 8.93 min was found to be the butenolide tertiary alcohol **2**. The previously characterized compounds **2** and **3** were first isolated by Mukku *et al.* and they suggest that the precursor of butenolide alcohols **1a**, **1b** and **2** is an epoxide [15]. To investigate if a precursor for these isomers could be detected and isolated, a new culture was prepared using the same conditions as above. Samples were taken every hour between 40 and 48 h of cultivation. Supernatant and bacteria were extracted separately with EtOAc and analyzed with reversed-phase HPLC after concentration. Interestingly, we detected a peak at retention time 13.50 min after 46 h of cultivation with similar UV-vis profile as butenolides **1**–**3** (Figure S2 in Supplementary Information) from the bacterial extract. At this time point, the butenolide alcohols **1a**, **1b** and **2** were not detected. The peak at 13.41 min was isolated and shown to be the butenolide **4** with a non-functionalized side-chain. Butenolide **4** was primarily found in the bacteria in contrast to butenolide alcohols **1a**, **1b** and **2** that were mostly present in the supernatant. However, we could not under these conditions detect an epoxide precursor. Therefore, we postulate that the precursor is the butenolide **4** with a non-functionalized side-chain. Compound **4** has not previously been isolated from actinobacteria, however, it has been detected by mass spectroscopy and been synthesized without stereospecificity [16].

#### 2.2.2. Cultivation of *Streptomyces* sp. AW28M48 and Isolation of Butenolide Analogs

Inoculated media were cultivated for two and four days, respectively, at 26 °C and 160 rpm. After cultivation, the bacteria were separated from the broth and extracted separately. The bacterial pellet from two days of cultivation was mixed with H_2_O and extracted with EtOAc. The resulting residue was mixed with hexane and filtrated through glass wool to remove an orange-yellow precipitate containing mostly flavanoids. After concentration the residue was fractionated over silica gel using a flash column followed by reversed phase chromatography to yield the butenolide **4** with a non-functionalized side-chain, as a colorless oil. Supernatants from two and four days, respectively, were extracted twice with EtOAc at pH 8. The flavanoid compounds, that complicated the purification of butenolide tertiary alcohol **2**, were precipitated off with hexane. Flash chromatography over silica gel followed by reversed phase chromatography gave the butenolide tertiary alcohol **2**, secondary alcohol **1a** and **1b** and ketone **3** as colorless oils. The structures of butenolides **1**–**4** were confirmed by NMR spectroscopy, HRMS, CD and optical rotation measurements and these were compared with published data [15,17,18,19].

#### 2.2.3. Structure Elucidation of the Butenolide Analogs

The diastereomeric mixture of the butenolide **1a** and **1b** was first isolated from a *Streptomyces* sp. by Mukku *et al.* in 2000 [15]. From ^13^C and ^1^H-NMR data it was believed to be a 1:1 mixture of two inseparable diastereomers. The absolute configuration for C-4 could be determined to be S from comparison of the CD-spectra with the absolute stereochemistry of a known butenolide. Later, total synthesis of the four possible stereoisomers was achieved by Karlsson *et al.* 2007, in order to solve the absolute configuration of the remaining two stereogenic centers C-10 and C-11 [17]. From comparison of the ^13^C-NMR data with the published data by Mukku *et al.* [15], it was shown that the stereo configuration should be [4_S_10_R_11_S_ or 4_S_10_S_11_R_] and [4_S_10_R_11_R_ or 4_S_10_S_11_S_]. Additional optical rotation data for the synthetic isomers published by Wang *et al.* in 2010 revealed that the two naturally occurring butenolide secondary alcohols were most likely 4_S_10_R_11_S_ and 4_S_10_R_11_R_ [19]. In this work, we isolated butenolide secondary alcohols **1a** and **1b** from two different cultivation times with different diastereomeric excesses. The ratio between the two diastereomers of butenolide secondary alcohols **1a** and **1b** was determined from H-NMR by comparing the integral of the C-11 proton. After two days, the ratio of the diastereomeric mixture was 12:1 in favor of butenolide **1a**, in comparison to a 1:1 mixture after the four days cultivation. Optical rotation data (Table 1) and ^13^C-NMR of the butenolide secondary alcohols **1a** and **1b** (Table 2), isolated after two days, match the reported data by Wang *et al.* for the synthetic butenolide secondary alcohol **1a** (4_S_10_R_11_S_). Therefore, we conclude that the absolute stereochemistry for the natural butenolide secondary alcohol **1a** is 4_S_10_R_11_S_ and hence 4_S_10_R_11_R_ for **1b**. ^13^C-NMR data for our isolated butenolides **1**–**4**, are consistent with previously reported data (Table 2). Also positive CD absorption, for all isolated butenolides, at 204–208 nm (π-π′) confirms *S*-configuration at C-4 [15]. Optical rotation data for compound **3** matches the data reported by Wang *et al.* for the absolute stereochemistry of 4_S_10_R_ [19].

**Table 1:** Optical rotation of butenolide **1**–**4** in MeOH.

Compound	Optical Rotations	Optical Rotations from Previous Publications
**1a** and **1b** (1:1 mixture)	+80.8° (*c* 1.15, MeOH)	+84.5° (*c* 0.119, MeOH)(natural) [15]
**1a** (4_S_10_R_11_S_)	+74.4° (*c* 0.926, MeOH) (85% *de*)	+70.9° (*c* 0.12, MeOH)(synth.) [17]
		+78° (*c* 0.1, MeOH)(synth.) [18]
**1b** (4_S_10_R_11_R_)		+64.3° (*c* 0.14, MeOH)(synth.) [19]
**2**	+66.5° (*c* 0.927, MeOH)	+44° (*c* 0.072, MeOH)(natural) [15]
**3** (4_S_10_R_)	+48.8° (*c* 1.475, MeOH)	+45° (*c* 0.119, MeOH)(natural) [15]
		+49.4° (*c* 0.175, MeOH)(synth.) [19]
**3** (4_S_10_S_)		+73.0° (*c* 0.12, MeOH)(synth.) [19]
**4**	+58° (*c* 1.033, MeOH)	

**Table 2:** ^13^C-NMR chemical shifts (ppm) of butenolide **1**–**4** in CDCl_3_.

Position	1a	1a [17]	1a + (1b)	1b [19]	2	2 [15]	3	3 [19]	4	4 [16]
1 CO	173.12	173.19	173.12	173.14	173.1	173.2	173.07	173.09	173.2	173.1
2 CH	121.57	121.54	121.57	121.54	121.6	121.6	121.59	121.59	121.5	121.5
3 CH	156.20	156.30	156.3	156.23	156.2	156.2	156.16	156.17	156.3	156.3
4 CH	83.36	83.41	83.36	83.39	83.3	83.4	83.29	83.29	83.4	83.4
5 CH_2_	33.16	33.15	33.16	33.15	33.1	33.1	33.05	33.06	33.2	33.2
6 CH_2_	24.94	24.95	24.96	24.95	25.0	25.0	24.79	24.79	25.0	25.0
7 CH_2_	29.60	29.60	29.60	29.60	29.9	29.9	29.30	29.30	29.6	29.6
8 CH_2_	26.97	26.98	27.11	27.10	23.6	23.6	26.94	26.96	26.8	26.8
9 CH_2_	32.33	32.34	32.43	32.41	41.1	41.1	32.62	32.62	36.4	36.4
10	39.98 CH	39.97 CH	39.68 CH	39.67 CH	72.8 C_q_	72.9 C_q_	47.10 CH	47.11 CH	34.3 CH	34.3 CH
11	71.71 CH	71.70 CH	71.29 CH	71.30 CH	34.3 CH_2_	34.3 CH_2_	212.77 CO	212.80 CO	29.4 CH_2_	29.4 CH_2_
12 CH_3_	19.51	19.49	20.26	20.25	8.2	8.2	28.01	28.03	11.4	11.3
13 CH_3_	14.58	14.57	14.12	14.11	26.4	26.4	16.28	16.29	19.2	19.1

### 2.3. Proposed Biosynthetic Pathway of Butenolide **1**–**4**

**Figure 4:**
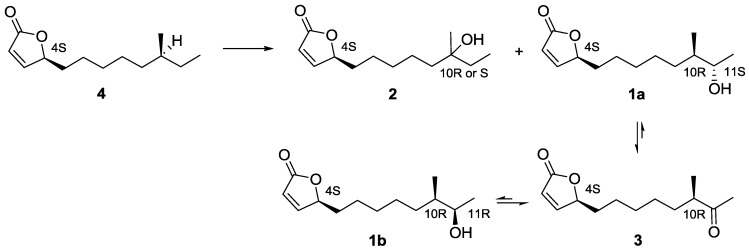
The proposed biosynthetic pathway of butenolides **1**–**4**.

From the time of appearance and structure of the butenolides **1**–**4**, we propose that the butenolide **4** with a non-functionalized side chain is the starting precursor for the biosynthesis of butenolides **1**–**3**. We believe that the side chain in butenolide **4** is oxidized stereo-specifically to one of the two possible diastereomers of the tertiary alcohols **2** (4_S_10_R_) or **2** (4_S_10_S_) and the secondary alcohol **1a** in favor of the latter. If this hypothesis is correct, the absolute stereochemistry for the butenolide **4** with a non-functionalized side chain should be 4_S_10_S_. We also propose that the butenolide secondary alcohol **1a** is oxidized to the butenolide ketone **3**, and during this process epimerization of the hydroxyl at C-11 by unspecific reduction or via reverse oxidation of the ketone results in a 1:1 ratio of the secondary alcohols **1a** and **1b** (Figure 4). The enzyme(s) responsible for the oxidation in the biosynthetic pathway are currently under investigation.

### 2.4. Biological Evaluation of Butenolide Analogs

#### 2.4.1. The Anti-Adenoviral Activity of the Different Butenolide Analogs **1**–**4**

**Figure 5:**
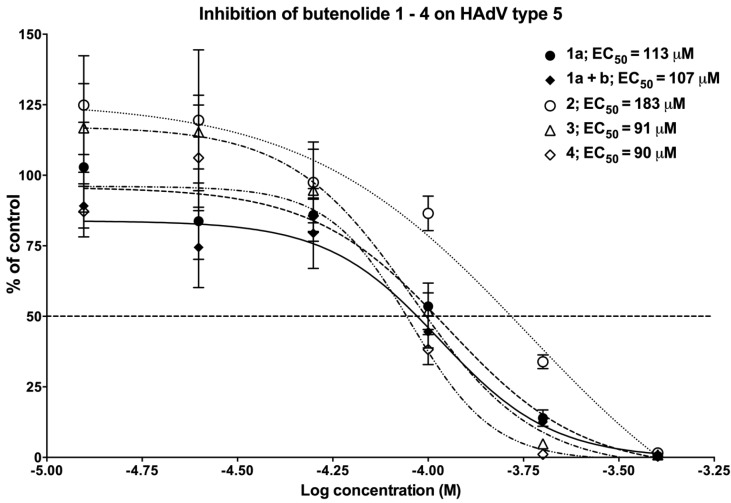
Dose-response study of the identified compounds on HAdV type 5. Each concentration point was run in duplicate and qPCR-analysis performed after 24 h. The amount of HAdV type 5 genomes in each treated sample was compared to infected but non-treated samples to obtain the inhibition of the percentage of control (*y*-axis). See the experimental section for details.

In order to confirm the antiviral activity observed during the screening campaign (Figure 3) and determine the EC_50_ values of the analogs, we conducted a dose-response experiment by a standardized quantitative real-time PCR (qPCR) assay (Figure 5). In this assay, the number of adenoviral genomes is assessed in relationship to the number of cells and the replication of HAdV type 5 on A549 host cells was assessed. Small differences were observed in the potency of the analogs (Figure 5). The activity of butenolide tertiary alcohol **2**, with an EC_50_ value of 183 μM was lower than the other analogs, which had similar EC_50_ values, ranging from 90 to 113 μM. Notably, the activity of the diastereomeric mixture of **1a** with **1b** did not differ much from **1a** alone, with EC_50_ values of 107 and 113 μM, respectively, indicating that the stereochemistry of the hydroxyl group is of low importance for antiviral activity. In conclusion, the butenolide ketone **3** and the butenolide **4** with a non-functionalized side-chain were the most potent analogs, with almost identical EC_50_ values, and these were selected for further analysis.

#### 2.4.2. The Two Most Potent Analogs, Butenolides **3** and **4**, Possess Similar Antiviral Activity but Differ Widely in Cytotoxicity

**Figure 6:**
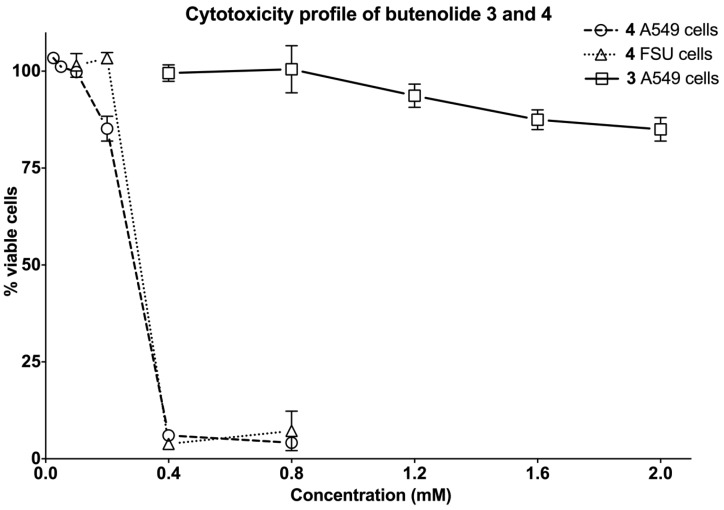
The cytotoxicity profile of butenolide ketone 3 and butenolide 4 with a non-functionalized side chain, on two cell lines, A549 cells and FSU cells, analyzed after 24 h. See experimental section for details.

Consideration of cellular cytotoxicity is essential when identifying antiviral compounds. Compounds that negatively interfere with the proliferation or viability of host cells will be recorded as false positives when assayed for antiviral activity. Based on the morphology of the host cell nuclei, the extract from the actinobacteria containing the butenolide compounds displayed no apparent cytotoxicity (Figure 3). To confirm these data, we performed a XTT based cytotoxicity test at approximately four and eight times the average EC_50_ concentration of the compounds. The XTT test measures the ability of mitochondrial enzymes to reduce 2,3-bis-(2-methoxy-4-nitro-5-sulfophenyl)-2*H*-tetrazolium-5-carboxanilide to a colored formazan dye that is spectrophotometrically quantified. Table 3 shows the percentage viable A549 cells compared to untreated DMSO control after 24 h incubation with compound. Strikingly, butenolide **4** with the non-functionalized side-chain affected the viability dramatically. At both 400 and 800 μM of **4** the percentage of viable cells was close to zero, whereas for butenolide **1**–**3** the cells were as viable as the DMSO control at the concentrations tested. The butenolide ketone **3** and the butenolide **4** with a non-functionalized side-chain were the most potent compounds with a similar EC_50_ value of 91 and 90 μM, respectively, but their cytotoxicity at 400 and 800 μM differed widely. To further elucidate this difference and determine whether the cytotoxicity was cell type specific, we performed a dose-response experiment and investigated the cytotoxicity on human diploid fibroblasts derived from foreskin (FSU). FSU cells are isolated from the dermis of juvenile foreskin and, unlike A549 cells, are not derived from carcinoma, but primary cells with a limited expected number of cell divisions. The butenolide ketone **3** did not affect the viability of FSU cells to a great extent, even at the highest concentration of 2 mM (Figure 6). However, the butenolide **4** with a non-functionalized side-chain reduced the viability of A549 cells and FSU cells to almost zero at 400 and 800 μM. This is consistent with the data in Table 3, but the toxicity was strongly decreased at 200 μM and at 100 μM the viability is in line with untreated DMSO control. Together with the EC_50_ value of butenolide **4**, 90 μM, this showed that the difference in concentration between cytotoxicity and antiviral activity is small. The reason for the steep concentration dependence on cytotoxicity for the butenolide **4** containing a non-functionalized side-chain is presently unknown, and the large difference in cytotoxic potential between **4** and the other analogs is noteworthy when considering the minor structural differences in their side-chains.

**Table 3:** The cytotoxicity of the identified compounds at two fixed concentrations on A549 cells after 24 h based on two independent experiments. The values were normalized against DMSO controls, with corresponding amounts of added DMSO at 400 and 800 μM.

Compound	% Viable Cells at 400 μM	% Viable Cells at 800 μM
**1a**	98 ± 6	96 ± 4
**1a** + **1b**	105 ± 8	98 ± 1
**2**	106 ± 7	100 ± 3
**3**	102 ± 5	101 ± 3
**4**	3 ± 1	3 ± 1

**Figure 7:**
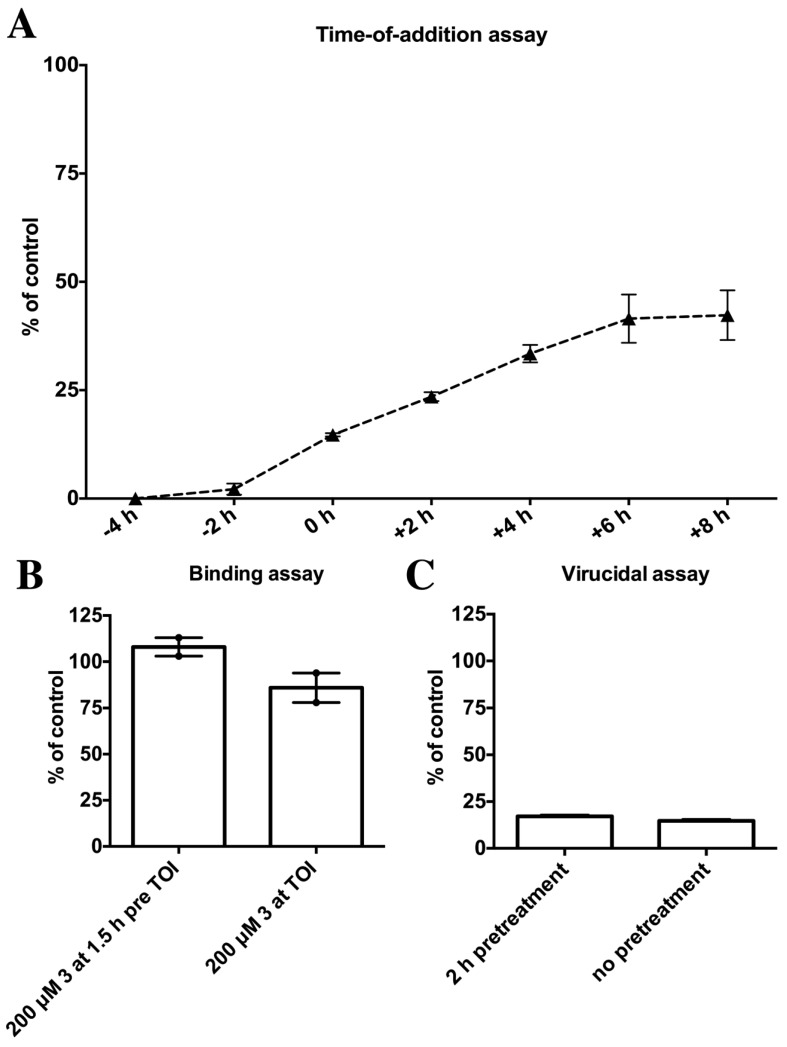
Characterization of the antiviral activity of the butenolide ketone **3**. The antiviral activity of the compounds was obtained by comparing the amount of HAdV type 5 genomes per cell to non-treated but infected control samples (*y*-axis). (**A**) Time-of-addition assay where 200 μM of butenolide ketone **3** was added at different time points in comparison to the addition of HAdV 5 at time 0 h. Samples were analyzed 24 h after the infection (+24 h) with qPCR; (**B**) Binding assay with ^35^S-labelled HAdV 5 virions, with 200 μM compound **3** pretreated A549 cells for 1.5 h prior time-of-infection (TOI) and no pretreatment; (**C**) Virucidal assay where HAdV 5 virions were pretreated with 200 μM of butenolide ketone **3** prior addition to A549 cells and analyzed after 24 h.

#### 2.4.3. Characterization of the Antiviral Activity of Butenolide Ketone **3**

Since the butenolide **4** with a non-functionalized side-chain already displayed cytotoxicity at approximately three times its EC_50_ value, we selected the butenolide ketone **3** for which we observed no cytotoxicity and the lowest EC_50_ value, for further characterization of the antiviral activity. This was primarily to investigate in which stage of the viral infectious cycle that the butenolide ketone **3** inhibited and consequently to reduce the number of possible drug-targets for further mode-of-action analysis. Figure 7A shows the time-of-addition assay where 200 μM of butenolide ketone **3** was added at different time points, in relation to the addition of HAdV type 5. Simultaneous addition of 200 μM of **3** and virus (0 h) reduced the viral replication to below 20%, which is consistent with Figure 5. However, pre-incubation of the cells with 200 μM of **3** for four hours prior to the addition of virus completely blocked the viral infection. Since the data is based on the qPCR assay that measures the amount of newly synthesized viral genomes, a complete blockage when cells are pre-treated could indicate that the inhibitory step is prior to the replication of the viral genomes. Such early viral events are viral attachment, viral uptake and viral transportation to the nuclear pore. To elucidate if the butenolide ketone **3** inhibited the viral binding to the cell we conducted a binding assay with radiolabeled HAdV 5 virions (Figure 7B). However, cells that were pre-treated with 200 μM of butenolide ketone **3** did not reduce the binding of the virus to the cells as compared to the control. Furthermore, to investigate the possibility that the butenolide ketone **3** could have virucidal activity, e.g., have affinity to viral structural proteins and thereby inhibits viral replication, we incubated HAdV 5 virions together with 200 μM of butenolide **3** for 2 h, infected the cells and evaluated the amount of replicated viral genomes present after 24 h (Figure 7C). However, the levels of inhibition observed did not differ between the pretreated and non-pretreated virions. Conclusively, the complete blockage of viral replication when the cells were pretreated with the butenolide ketone **3** indicates that **3** may act on a cellular target or a cellular process that is essential for viral replication and the addition of compound prior to virus would enhance the antiviral effect. Thus, a prophylactic administration in a clinical setting would preferably prevent the viral infection, or if administrated later, butenolide ketone **3** would inhibit the spread of the infection to non-infected cells. However, further investigations into the precise mode-of-action of butenolide ketone **3** are needed.

#### 2.4.4. Reduction of the 2-Furanone Moiety Eliminates the Antiviral Activity

**Figure 8:**
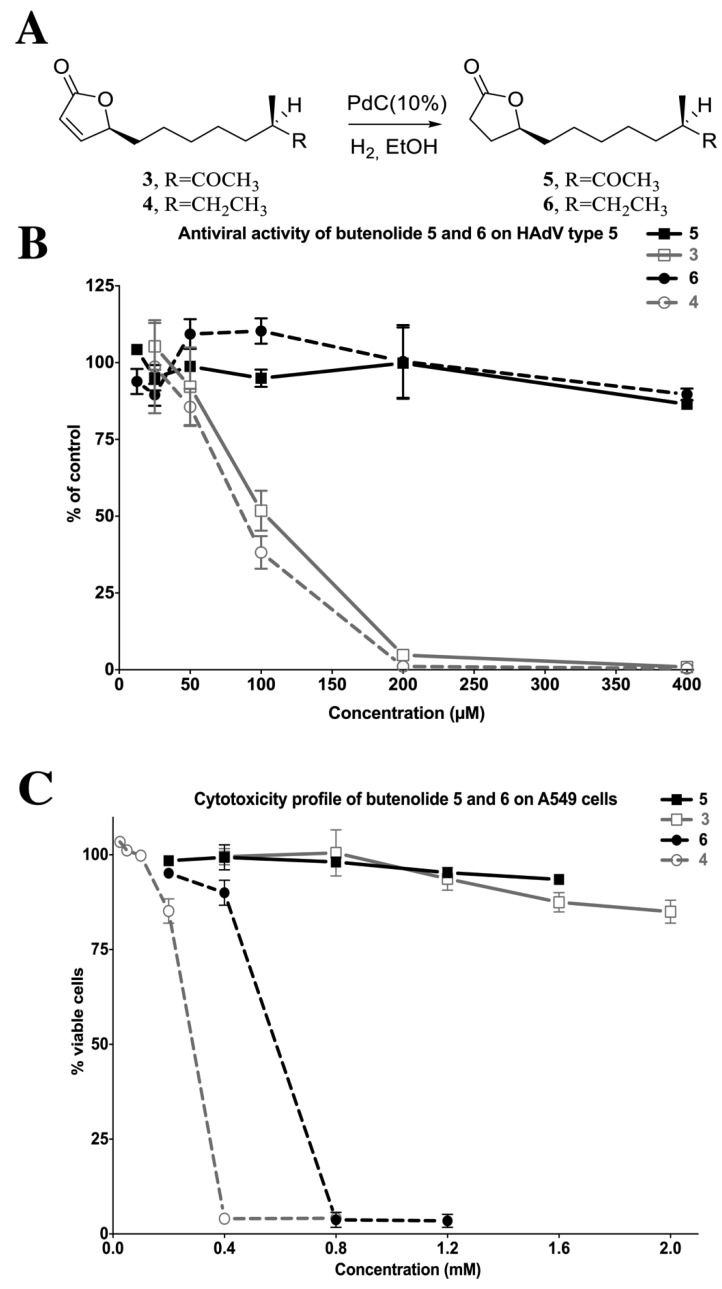
The structure and bioactivity of compound **5** and **6** in comparison with compound **3** and **4**. (**A**) Reduction of the double bond in the 2-furanone moiety of compound **3** and **4** generated compounds **5** and **6**; (**B**) The anti-adenoviral activity of **5** (black squares) and **6** (black circles) in comparison to **3** (grey open squares) and **4** (gray open circles), analyzed 24 h post infection with qPCR. The antiviral activity of the compounds was obtained by calculating the amount of HAdV type 5 genomes per cell to non-treated but infected control samples (*y*-axis); (**C**) The cytotoxicity profile of **5** (black squares) and **6** (black circles) in comparison to **3** (grey open squares) and **4** (gray open circles). The cytotoxicity was assessed on A549 cells after 24 h incubation with the compounds using the XTT-based kit. See the experimental section for details.

Considering that variation in the side-chains of butenolide **3** and **4** resulted in notable differences in the cytotoxic potential (Figure 6) but not in the antiviral potency (Figure 5), we performed a further structure-activity analysis to elucidate the significance of the 2-furanone moiety as the functional group responsible for antiviral activity. By removing the functionality of the Michael acceptor by reduction of the double bond in the 2-furanone moiety of **3** and **4**, compounds **5** and **6** were obtained (Figure 8A). To assess the bioactivity of **5** and **6**, we performed dose-response experiments and evaluated the antiviral activity and cytotoxicity (Figure 8B). Reduction of the double bond eliminated the antiviral activity for both butenolide ketone **3** (compound **5**) and butenolide **4** with a non-functionalized side-chain (compound **6**). It has previously been suggested that the double bond is responsible for the anti-fouling activity of the barnacle *Balanus amohitrite* and that removal or a shift in position would eliminate the bioactivity [14]. However, according to Figure 8C, the cytotoxicity is still present for compound **6**, though it is reduced compared to butenolide **4**. Considering the concentrations tested, compound **6** significantly reduces the viability of the cells above 0.4 mM, whereas compound **4** was comparably cytotoxic above 0.2 mM, and the concentration endpoint in Figure 8B where no antiviral effect is observed is 0.4 mM. This corresponds to the previous report [14], which stated that the bioactivity against barnacles was abolished at the maximum tested concentration (0.47 mM). Although, based on Figure 8A,B, the bioactivity cannot solely be attributed to the double bond in the 2-furanone, since compound **6** displayed cytotoxicity at higher concentrations, but we can conclude that the double bond is of importance for the antiviral activity. Furthermore, the absence of the ketone group in compound **4** increases its lipophilicity in comparison to compound **3**. Theoretically, this lipophilicity could be associated with the cytotoxic potential, e.g., the intracellular concentration of compound **4** could become higher than the concentration of **3** due to increased diffusion through cell membrane. However, this is contradictory since **3** and **4** have similar EC_50_ values against adenovirus (Figure 5). The mode of action for antiviral activity of compound **3** is currently not known but the mode of action for antifouling and antiproliferative activity for compound **4**, or similar compounds has been suggested depending on organism and cell [16,20,21]. Zhang *et al.* in 2011 [20] reported that the antifouling butenolide compound induced apoptosis in zebrafish and HeLa cells by activation of Bcl-2 proteins and c-Jun *N*-terminal kinases, and later reported a direct binding and inhibition of enzymes essential for the primary metabolism of marine target organisms. Furthermore, a 2-furanone compound with only a ketone group as side-chain was shown to deplete the intracellular glutathione depot in HepG2 cells, and the parent 2-furanone heterocycle without any side-chain showed DNA damaging activity and inhibition of the formation of topoisomerase and DNA complexes in A549 cells and MRC5 fibroblasts [22,23]. Nonetheless, the precise antiviral mode of action for compound **3**, in particular, is to be assessed further.

## 3. Experimental Section

### 3.1. General Experimental Procedures

Optical rotations were measured at 589 nm on a 343 polarimeter (Perkin-Elmer, Waltham, MA, USA) at 20 °C. CD spectra were recorded on a J-810 spectropolarimeter (Jasco Corporation, Cremella, Italy) [λ([θ]) in nm]. H^1^ and C^13^ NMR spectra were recorded on a DRX-400 spectrometer (Bruker, Billerica, MA, USA) at 298 K. ^1^H and ^13^C chemical shifts are reported relative to CHCl_3_ (δ_H_ 7.26 ppm) or CDCl_3_ (δ_C_ 77.0 ppm) as internal reference. ^13^C chemical shifts for compounds **1a**, **1b** and **3** is described with two decimals for comparison with previously published data containing two decimals. High resolution mass spectra (HRMS) data was recorded with micrOTOF II (Bruker, Billerica, MA, USA). TLC was performed on silica gel 60 F_254_ (Sigma-Aldrich, St. Louis, MO, USA) with detection of UV light and anisaldehyde-sulfuric acid as spray reagent. Flash Column chromatography [eluent for flash chromatography is given between brackets in the Experimental Section] was carried out on silica gel (particle size, 60 Å, 230–400 mesh) Sigma-Aldrich. Preparative HPLC was performed using VP 150/10 Nucleodur C-18, HTEC, 5 µm column (Macherey-Nagel, Düren, Germany) on a Gilson 333/334 Prep-Scale system with a flow rate of 7 mL/min, detection at 210 nm Gilson 151 (Gilson, Middleton, WI, USA), and an CH_3_CN (0.005% HCO_2_H)/H_2_O (0.005% HCO_2_H) eluent system. Analytical HPLC was performed using EC 150/4.6 Nucleodur C18 HTec, 5 µm column (Macherey-Nagel, Düren, Germany) on a Gilson 322 with a diode array detector Gilson 172 and evaporated light scattering detector Sedex 85 (Sedere, Oilvet, France). Reductions were performed using a H-Cube (ThalesNano, Budapest, Hungary) continuous-flow hydrogenation system utilizing a 10% Pd/C CatCart catalyst at 1 bar pressure with a flow rate of 1 mL/min at 20 °C. Cultivation of bacteria was carried out in 500 mL Erlenmeyer flasks with one baffle (Glasgerätebau OCHS, Bovenden, Germany).

### 3.2. Isolation of Actinobacteria from the Arctic Sea

Various biota and sediment samples were obtained during a research-cruise with R/V Jan Mayen in Vestfjorden, Northern Norway in April 2010. The samples were used as inoculum on different selective agar plates as described [24]. On land the bacterial plates were further incubated at 4–16 °C for up to one month. Single colonies were re-streaked onto new plates and colonies from these were used to inoculate 5 mL cultures kept at 16 °C. One mL of dense culture was cryopreserved in 20% glycerol at −80 °C. For sequencing, 1 mL of culture was harvested by centrifugation using a table-top centrifuge at 12,000 rpm for 3 min, washed once with 1 mL of distilled water and re-centrifuged for 3 min. Instagene matrix (BioRad, Hercules, CA, USA) was used to extract genomic DNA according to the manufacturer’s procedures. The universal primers 27F (5′-AGAGTTTGATCCTGGCTCAG-3′) and 1492R (5′-TACGGYTACCTTGTTACGACTT-3′) were used in standard PCR to obtain a fragment of the 16s rRNA gene. PCR product was purified using PureLink Pro 96 PCR Purification Kit (Invitrogen-Life Technologies, Carlsbad, CA, USA) following manufacturer’s protocols. Further, the sequencing primer 515F (5′-GTGCCAGCAGCCGCGGTAA-3′) was used in the sequencing PCR following the BigDye Terminator v3.1 Cycle Sequencing Kit protocol (Applied Biosystems, Carlsbad, CA, USA). The additional handling was done at the University of Tromsø’s DNA sequencing core facility. The ABI2FASTA converter v 1.1.2 [25] was used to extract FASTA sequence files from ABI output files and low quality ends were trimmed [26]. The trimmed sequences were then checked for chimeras using DECIPHER’s Find Chimeras web tool [27]. Sequence search against GenBank using BLAST was performed to identify the genus each bacterium belongs to [28]. In total, 57 strains of actinobacteria were isolated.

### 3.3. Extract Library

The 57 actinobacterial strains were re-streaked on M4 agar plates (0.1% malt extract, 0.1% glycerol, 0.1% glucose, 0.1% peptone, 0.1% yeast extract, 2% sea salts (Sigma Aldrich #S9883, St. Louis, MO, USA) and 20% agar in distilled water, pH was adjusted to pH 8.2) and incubated at 4 °C until growth was observed, approximately one week, there-after the plates were stored at 4 °C [24]. Colonies from single cells were inoculated in liquid M4 medium and incubated for two days at 26 °C with shaking (160 rpm). One milliliter of the pre-cultures were added to 120 mL of four different production media (PM1-4) with or without the addition of 4% sea salts yielding eight different production media. PM1 contains 1% glucose, 2% soluble starch, 0.3% Bacto peptone, 0.3% meat extract, 0.5% yeast extract and 0.3% CaCO_3_ in deionized water, pH was adjusted to 7 prior to sterilization, PM2 contains 1% glucose, 1% glycerol, 0.5% oat meal, 1% soy meal, 0.5% yeast extract, 0.5% bacto casaminoacids and 0.1% CaCO_3_ in deionized water, pH was adjusted to 7 prior to sterilization, PM3 contains 1% starch, 1% glucose, 1% glycerol, 0.25% corn steep powder, 0.5% peptone, 0.2% yeast extract, 0.1% NaCl and 0.3% CaCO_3_ in deionized water, pH was adjusted to 7.3 prior to sterilization [29] and PM4 contained 1.7% tryptone, 0.3% soytone, 1% glucose, 0.5% NaCl, 0.25% K_2_HPO_4_ in deionized water, pH was adjusted to 7.3 prior to sterilization. The production media were incubated for five days at 26 °C with shaking (160 rpm). At day five, the cultures were centrifuged at 3700 rpm for 10 min to pellet the bacteria, the supernatants were divided into two aliquots and pH was adjusted to 5 and 8, respectively. 1:1 EtOAc was added to the supernatants and mixed by vigorous shaking for one minute. The phases were separated by centrifugation at 3700 rpm for five minutes and the EtOAc phases were evaporated. The residues were re-dissolved in 1 mL DMSO and placed in 96-well plates. In total 912 extracts were yielded. Media controls were added to all 96-well plates.

### 3.4. *Streptomyces* sp.

The strain was isolated at: N67 52.11512 E16 22.97003 at a depth of 417 m on 17 April 2010. It was isolated from a sediment sample collected with a Van Veen grab sampler. This strain is preserved and available from an in-house collection at the University of Tromsø, Norway (database index AW28M48). The 16s rRNA gene sequence has been determined and is deposited within European Nucleotide Archive (accession number: HG764583). A phylogenetic analysis has been made based on the 16s rRNA gene sequence (Figure S18 in Supplementary Information).

### 3.5. Fermentation

Bacteria were pre-cultured in M4 medium for approximately 48 h. One and a half milliliters of the pre-cultured bacteria was added to 200 mL PM2 with 4% sea salt. The bacterial cultures were grown for two to five days at 26 °C, 160 rpm in a rotary shaker incubator. At different time points, the bacteria were pelleted at 3200 rpm for 20 min. For scale-up experiments, the chosen number of flasks was multiplied according to the volume needed.

### 3.6. Bioactivity Guided Fractionation

The obtained EtOAc extract from a 200 mL culture was re-dissolved in 300 μL DMSO. One hundred and fifty microliters of the re-dissolved extract was fractionated using preparatory HPLC (Nucleodur C_18_ HTec, 110 Å/5 µm, 10 × 160 mm) using a gradient from 10% to 100% CH_3_-CN/H_2_O (0.005% HCO_2_H) over 14 min followed by 100% CH_3_-CN (0.005% HCO_2_H) for 14 min. A total of 130, 1.5 mL fractions were collected at a flow-rate of 7 mL per min. After lyophilization, fractions were dissolved in 50 μL DMSO and transferred to 96-well plates and analyzed for anti-adenoviral activity. Active fractions were further purified to single compound entities with reverse-phase HPLC.

### 3.7. Extraction and Isolation of Secondary Metabolites from Marine Actinobacteria

The pH of the supernatant from 5 L broth (PM2 with 4% sea salt) was adjusted to pH 8 using 1 M NaOH (aq) and extracted twice with EtOAc, 1:1 v:v. After centrifugation, the organic layer was concentrated under vacuum that gave a residue of 1.8 and 2.2 g for the 48 and 96 h culture, respectively. The bacterial pellet was dissolved in deionized water and extracted with EtOAc, 1:1 v:v. After centrifugation, the organic phase was concentrated which gave an oily residue of 8.5 g. The EtOAc extract of 48 h culture was mixed with hexane and filtrated through glass-wool to remove an orange precipitate. After concentration, the resulting residue was pre-purified with flash chromatography over silica gel [CHCl_3_:MeOH, 95:5]. Fractions 9–11 (197 mg) contained compounds **3**–**4**, fractions 12–14 (61 mg) contained compounds **1a**, **1b**, **2** and **3** and fractions 15–19 (36 mg) contained compounds **1a**, **1b**, and **2**. Fractions 9–11 was mixed with 450 μL DMSO and purified with preparatory HPLC (C_18_, Nucleodur HTEC, 110 Å/5 µm, 10 × 160 mm) using a gradient from 30% to 100% CH_3_-CN/H_2_O (0.005% HCO_2_H) during 10 min. Fractions containing compounds **3** and **4** were neutralized with NaHCO_3_ (aq) and extracted with CHCl_3_. Organic phases were concentrated in vacuum which gave 1.5 mg of compound **3** and 1 mg of compound **4** as colorless oils. In the same way, fractions 12–14 and 15–19 were purified using a gradient from 10% to 100% CH_3_-CN/H_2_O (0.005% HCO_2_H) during 40 min which gave 1.3 mg of compound **2**, 6 mg of compounds **1a** and **1b** (12:1) and 0.8 mg of compound **3**. The workup of EtOAc extract from 96 h culture was performed in the same way as the 48 h culture that gave 2.1, 5.8 and 8 mg of compounds **1a**, **1b**, **2** and **3**, respectively. The oily residue of the bacterial pellet was mixed with hexane and filtrated through a plug of glass wool to remove an orange precipitate. After concentration the resulting residue was pre-purified with flash chromatography over silica gel [Pentane:Et_2_O, 3:1]. Fractions containing compound **4** (34–59) was concentrated and mixed with 450 μL DMSO. The resulting mixture was purified using preparatory HPLC, seven injections (Nucleodur C_18_ HTec, 110 Å/5 µm, 10 × 160 mm) using a gradient from 50% to 100% CH_3_-CN/H_2_O (0.005% HCO_2_H) during 10 min. Fractions containing compound **4** were neutralized with NaHCO_3_ (aq) and extracted twice with CHCl_3_. The combined organic phases were concentrated in vacuum that gave 11.4 mg of compound **4** as a colorless oil.

**1a:** Isolated after 48h, [α]^22^_D_ +74.4° (*c* 0.93, MeOH); CD (*c* 0.110, MeOH) ∆_ε208_ +15; ^1^H-NMR (400 MHz CDCl_3_) δ 7.44 (*J*_1_ = 5.7, *J*_2_ = 1.4 Hz, 1 H), 6.11 (dd, *J*_1_ = 5.7, *J*_2_ = 1.9 Hz, 1 H), 5.06–5.01 (m, 1H), 3.68–3.61 (m, 1H), 1.83–1.72 (m, 1H), 1.72–1.61 (m, 1H) 1.59–1.17 (m, 9H), 1.13 (d, *J* = 6.34 Hz, 3H), 0.86 (d, *J* = 6.69 Hz, 3H); ^13^C-NMR data of **1a** are listed in Table 1. HRMS (M + Na^+^) calculated for C_13_H_22_O_3_Na: 249.1467; found, 249.1466.

**1a and 1b (1:1 mixture):** Isolated after 96 h, [α]^22^_D_ +80.8° (*c* 1.15, MeOH), CD (*c* 0.115, MeOH) ∆_ε208_ +19; ^1^H-NMR (400 MHz CDCl_3_) δ 7.44 (*J*_1_ = 5.7, *J*_2_ = 1.4 Hz, 1 H), 6.10 (dd, *J*_1_ = 5.7, *J*_2_ = 1.9 Hz, 1 H), 5.06–5.01 (m, 1H), 3.74–3.67, 3.67–3.60 (each m, 1H), 1.83–1.72 (m, 1H), 1.72–1.61 (m, 1H) 1.59–1.17 (m, 9H), 1.15 (d, *J* = 6.34 Hz, 3H), 1.12 (d, *J* = 6.34 Hz, 3H), 0.88 (d, *J* = 6.69 Hz, 3H), 0.86 (d, *J* = 6.69 Hz, 3H); ^13^C-NMR (100 MHz CDCl_3_) δ 173.3, 156.4, 121.7, 83.5, 71.9, 71.5, 40.1, 39.9, 33.3, 32.6, 32.5, 29.8, 27.3, 27.1, 25.12, 25.10, 20.4, 19.7, 14.7, 14.3; Subtracted ^13^C-NMR data of **1b** are listed in Table 1 as **1a** + (**1b**); HRMS (M + Na^+^) calculated for C_13_H_22_O_3_Na: 249.1467; found, 249.1474.

**2:** Isolated after 48h, [α]^22^_D_ +66.5° (*c* 0.927, MeOH); CD (*c* 0.093, MeOH) ∆_ε205_ +16; ^1^H-NMR (400 MHz CDCl_3_) δ 7.44 (dd, *J*_1_ = 5.8, *J*_2_ = 1.5 Hz, 1 H), 6.11 (dd, *J*_1_ = 5.8, *J*_2_ = 2.0 Hz, 1 H), 5.05–5.00 (m, 1H), 1.84–1.72 (m, 1H), 1.72–1.61 (m, 1H), 1.53–1.40 (m, 6H), 1.40–1.31 (m, 4H), 1.14 (s, 3H), 1.11 (-OH broad singlett), 0.89 (t, *J* = 7.5 Hz, 3H); ^13^C-NMR data of **2** are listed in Table 1; HRMS (M + Na^+^) calculated for C_13_H_22_O_3_Na: 249.1467; found, 249.1470.

**3:** Isolated after 48h, [α]^22^_D_ +48.8° (*c* 1.475, MeOH); CD (*c* 0.147, MeOH) ∆_ε204_ +26; ^1^H-NMR (400 MHz CDCl_3_) δ 7.44 (dd, *J*_1_ = 5.7, *J*_2_ = 1.4 Hz, 1 H), 6.11 (dd, *J*_1_ = 5.7, *J*_2_ = 2.0 Hz, 1 H), 5.05–5.00 (m, 1H), 2.53–2.44 (m, 1H), 2.13 (s, 3H), 1.82–1.72 (m, 1H), 1.70–1.58 (m, 2H), 1.51–1.38 (m, 2H), 1.39–1.21 (m, 5 H), 1.08 (d, *J* = 7.4 Hz, 3 H); ^13^C-NMR data of **3** are listed in Table 1; HRMS (M + Na^+^) calculated for C_13_H_20_O_3_Na: 247.1310; found, 247.1310.

**4:** Isolated after 48h, [α]^22^_D_ +58° (*c* 1.033, MeOH); CD (*c* 0.103, MeOH) ∆_ε204_ +17; ^1^H-NMR (400 MHz CDCl_3_) δ 7.45 (dd, *J*_1_ = 5.7, *J*_2_ = 1.5 Hz, 1 H), 6.11 (dd, *J*_1_ = 5.7, *J*_2_ = 1.9 Hz, 1 H), 5.06–5.01 (m, 1H), 1.82-–1.62 (m, 2H), 1.51–1.38 (m, 2H), 1.38–1.21(m, 7H), 1.18–1.03 (m, 2H), 0.87–0.82 (m, 6H); ^13^C-NMR data of **4** are listed in Table 1; HRMS (M + Na^+^) calculated for C_13_H_22_O_2_Na: 233.1518; found, 233.1517.

**5:** Compound **3** (4.3 mg, 0.019 mmol) was dissolved in absolute EtOH and hydrogenated utilizing H-cube. The eluate was concentrated and co-concentrated with CHCl_3_ three times which gave 3.77 mg (0.017 mmol) as a colorless oil in 89% yield. [α]^22^_D_ −54° (*c* 0.76, MeOH); ^1^H-NMR (400 MHz CDCl_3_) δ 4.53–4.42 (m, 1H), 2.56–2.45 (m, 3H), 2.37–2.27 (m, 1H), 2.13 (s, 3H), 1.90–1.79 (m, 1H), 1.75–1.68 (m, 1H), 1.67–1.57 (m, 2H), 1.51–1.21 (m, 7H), 1.08 (d, *J* = 7 Hz, 3H); ^13^C-NMR (100 MHz CDCl_3_) δ 212.8, 177.2, 80.9, 47.1, 35.5, 32.7, 29.3, 28.8, 28.0, 28.0, 27.0, 25.1, 16.2; HRMS (M + Na^+^) calculated for C_13_H_22_NaO_3_: 249.1467; found, 249.1473.

**6:** Compound **4** (3.5 mg, 0.017 mmol) was dissolved in absolute EtOH and hydrogenated utilizing H-cube. The eluate was concentrated followed by Flash chromatography [3:1 pentane:Et2O] over silica gel to give 2.2 mg (0.010 mmol) as a colorless oil in 62% yield. [α]^22^_D_ −38° (*c* 0.79, MeOH); ^1^H-NMR (400 MHz CDCl_3_) δ 4.52–4.44 (m, 2H), 2.56–2.49 (m, 2H), 2.37–2.26 (m, 1H), 1.92–1.80 (m, 1H), 1.79–1.69 (m, 1H), 1.65–1.54 (m, 1H), 1.50–1.42 (m, 1H), 1.41–1.21 (m, 8H), 1.18–1.03 (m, 2H), 0.91–0.78 (m, 6H); ^13^C-NMR (100 MHz CDCl_3_) δ 177.3, 81.1, 36.5, 35.6, 34.3, 29.7, 29.4, 28.9, 28.0, 26.9, 25.2, 19.2, 11.4; HRMS (M+ Na^+^) calculated for C_13_H_24_NaO_2_: 235.1674; found, 235.1679.

### 3.8. Cells and Virus

A549 cells (human lung adenocarcinoma epithelial cells) and the diploid fibroblast cell line FSU (Foreskin Umeå, Division of Virology, Umeå, Sweden) were grown in DMEM (Sigma-Aldrich, St. Louis, MO, USA) containing 0.74 g of NaHCO_3_/L, 20 mM HEPES (Euroclone, Pero, Milano, Italy), 1× PEST and 5% fetal bovine serum (Gibco^®^, Life Technologies, Grand Island, NY, USA) at 37 °C. The RCAd11GFP vector used in the screening is a replication-competent Ad11 strain with a GFP insertion in the E1 region of the Ad11p genome [9] and the HAdV type 5 (strain F2853-5b) was used in the quantitative PCR assay.

### 3.9. Screening for Antiviral Activity in Actinobacteria Extracts

Approximately 10,000 A549 cells were seeded per well in 96-well plates (Nunc™ #167008, Thermo Fisher Scientific, Pittsburgh, PA, USA) on the day prior to extract addition. The following day, a pipetting robot **(**Biomek NX, Beckman, Brea, CA, USA**)** was used to dilute and add the extracts to the cells. The extracts were diluted 1:200 by mixing 1 μL of each extract in 100 μL phenol-red free DMEM with 2% FBS and 50 μL of mixture that replaced the growth media in the wells. Subsequently, 50 μL of phenol red free DMEM with 2% FBS containing low amounts of RCAd11GFP virus (0.025 pg per cell of RCAd11GFP) was added per well and the plates were incubated for 24 h at 37 °C. Thereafter, the nuclei of the cells were stained by adding 50 μL of Hoechst 33342 (Molecular Probes^®^, Life Technologies, Grand Island, NY, USA), diluted in PBS (0.2 μg/mL), to each well and the plates were incubated for 20 min, 37 °C, prior to analysis. The intensity of the GFP-expression per cell for each extract was analyzed in comparison to non-treated but infected cells using a Cellomics^®^ ArrayScanVTi^®^ (Thermo Fisher Scientific, Pittsburgh, PA, USA). In parallel, the primary cytotoxic assessment of the extracts was performed by visual examination of the nuclear morphology. Extracts that inhibited the GFP-expression with no or low impact on nuclear morphology was diluted for bioactivity confirmation.

### 3.10. Quantitative Real-time PCR for Antiviral Activity Confirmation

The quantitative Real Time PCR (qPCR) assay was performed as previously described [10,12]. Briefly, A549 cells in 24 well plates (Nunc™ #142475) were infected with HAdV type 5 (1 pg per cell) and incubated with compound for 24 h. Thereafter, cellular and viral DNA was prepared using a QIAamp DNA blood minikit (Qiagen, Solna, Sweden) and quantitative real-time PCR was used to detect newly synthesized viral DNA and the inhibitory effect of the compounds. The principle of quantitative real-time PCR and the design of primers and probes for analysis of various HAdV types representing different adenovirus species have previously been described [30,31]. Quantitative real-time PCR was carried out using a primer pair and a FAM-labeled probe detecting and amplifying the viral hexon gene. The amount of detected viral hexon DNA was normalized to the cellular RNaseP gene. Real-time PCR was performed in an ABI Prism 7900HT sequence detector and analyzed with Sequence Detector software version 2.4 (Applied Biosystems^®^, Life Technologies, Grand Island, NY, USA).

### 3.11. Cytotoxicity Assessment

The potential cytotoxic effect of the compounds was determined with XTT-based Cell Proliferation Kit II (Roche Applied Science, Penzberg, Germany). This method is a colorimetric assay based on the cleavage of the tetrazolium salt XTT to a soluble formazan salt by viable cells. Approximately, 13,000 A549 cells were seeded per well in 96-well plates on the day before addition of compounds. The next day, the growth medium was removed from the wells and replaced with 100 μL of phenol-red free DMEM with 2% FBS containing compound. In parallel, an amount of DMSO corresponding to the same amount of test compound was added to the cells and the plate was incubated at 37 °C for 20 h. Subsequently, 50 μL of XTT solution was added per well and the plate was incubated at 37 °C for 4 h and finally the intensity of the formazan dye was measured by spectrophotometry at a wavelength of 490 nm [32].

### 3.12. Binding Assay

^35^S labelling of HAdV 5 was performed as previously described [33] as well as the binding experiment [10]. In brief, A549 cells were detached with 0.05% EDTA in PBS and resuspended in growth media and were reactivated for 15 min at 37 °C. Next, 200 μM of compound **3** was added, and the cell suspension was incubated for 1.5 h at 37 °C. The cells were then dispensed in a 96-well plate and centrifuged at 1500 rpm, 5 min, at 4 °C. The pellets were resuspended in DMEM with 1% BSA and 200 μM **3**. ^35^S labelled HAdV 5 (1 pg per cell) was added and the plate was incubated for 1 h at 4 °C. Following, the cells were washed twice with ice-cold DMEM with 1% BSA and were transfered to scintillation tubes with 2 mL scintillation fluid (Optiphase HiSafe 3, Perkin-Elmer, Walltham, MA, USA). The cell-associated radioactivity was measured using a liquid scintillation counter (Wallac 1409, Perkin-Elmer, Waltham, MA, USA).

### 3.13. Virucidal Assay

The HAdV type 5 stock was diluted in DMEM (Sigma-Aldrich, St. Louis, MO, USA) with 2% FBS (Gibco^®^, Life Technologies, Grand Island, NY, USA) to the appropriate concentration to be added per well. Then 200 μM of the ketone butenolide **3** was added and the mixture was incubated at 37 °C for 2 h with shaking. Subsequently, the growth medium was removed from the cells and 500 μL of this mixture was added per well with confluent A549 cells in 24 well plates. In parallel, an identical mixture with no incubation time was added to wells as a control and the antiviral activity was analyzed 24 h post infection with qPCR assay.

### 3.14. Statistical Analysis

Determination of the EC_50_ values was performed with non-linear regression analysis with a variable slope using GraphPad Prism software version 6.0c (GraphPad Software, San Diego, CA, USA).

## 4. Conclusions

We have isolated the butenolide analogs **1**–**4** from marine actinobacteria and have characterized their anti-adenoviral activity. We demonstrate that the 2-furanone moiety in the structures is important for the anti-adenoviral activity, but not for the observed cytotoxicity. Furthermore, considering that the non-toxic butenolide ketone **3** is small and amenable to medicinal chemistry, it is an attractive starting point for further optimization of the anti-adenoviral activity.

## Supplementary Files

Supplementary File 1 Supplementary Information (PDF, 698 KB)
