# Supplementary material for: Isolation and Characterization of Anti-Adenoviral Secondary Metabolites from Marine Actinobacteria
Source: Mar Drugs. 2014 Jan 28;12(2):799–821. doi: 10.3390/md12020799 (PMC3944516; doi:10.3390/md12020799)
Supplement: Supplementary File 1 — Supplementary Information (PDF, 698 KB) [file marinedrugs-12-00799-s001.pdf]

Supplementary Information

Figure S1. Ethyl acetate extract of media supernatant after different time of cultivation.
Figure S2. Ethyl acetate extract of bacterial pellet after different time of cultivation.
Figure S3. UV-vis spectra of butenolides **1**–**4**.
Figure S4. ^{1}H-NMR (400 MHz, $CDCl_3$) spectrum of butenolide secondary alcohol **1a**.
Figure S5. ^{1}H-NMR (400 MHz, $CDCl_3$) spectrum of butenolide secondary alcohols **1a** and **1b** (1:1).
Figure S6. ^{1}H-NMR (400 MHz, $CDCl_3$) of spectrum of butenolide tertiary alcohol **2**.
Figure S7. ^{1}H-NMR (400 MHz, $CDCl_3$) spectrum of butenolide ketone **3**.
Figure S8. ^{1}H-NMR (400 MHz, $CDCl_3$) spectrum of butenolide non functional side chain **4**.
Figure S9. ^{1}H-NMR (400 MHz, $CDCl_3$) spectrum of saturated butenolide ketone **5**.
Figure S10. ^{1}H-NMR (400 MHz, $CDCl_3$) spectrum of saturated butenolide non functional side chain **6**.
Figure S11. ^{13}C-NMR (100 MHz, $CDCl_3$) spectrum of butenolide secondary alcohol **1a**.
Figure S12. ^{13}C-NMR (100 MHz, $CDCl_3$) of spectrum of butenolide secondary alcohols **1a**–**b**.
Figure S13. ^{13}C-NMR (100 MHz, $CDCl_3$) spectrum of butenolide tertiary alcohol **2**.
Figure S14. ^{13}C-NMR (100 MHz, $CDCl_3$) spectrum of butenolide ketone **3**.
Figure S15. ^{13}C-NMR (100 MHz, $CDCl_3$) spectrum of butenolide with non functional side chain **4**.
Figure S16. ^{13}C-NMR (100 MHz, $CDCl_3$) spectrum of saturated butenolide ketone **5**.
Figure S17. ^{13}C-NMR (100 MHz $CDCl_3$) spectrum of saturated butenolide **6**.
Figure S18. Phylogenetic analysis of *Streptomyces* sp. AW28M48.

Figure S1. Ethyl acetate extract of media supernatant after different time of cultivation: (**a**) 1 day; (**b**) 2 days; (**c**) 3 days; (**d**) 4 days. Retention times for butenolides **1**–**4**: **1a** and **1b** = 9.04 min; **2** = 8.93 min; **3** = 9.55 min; **4** = 13.50 min.

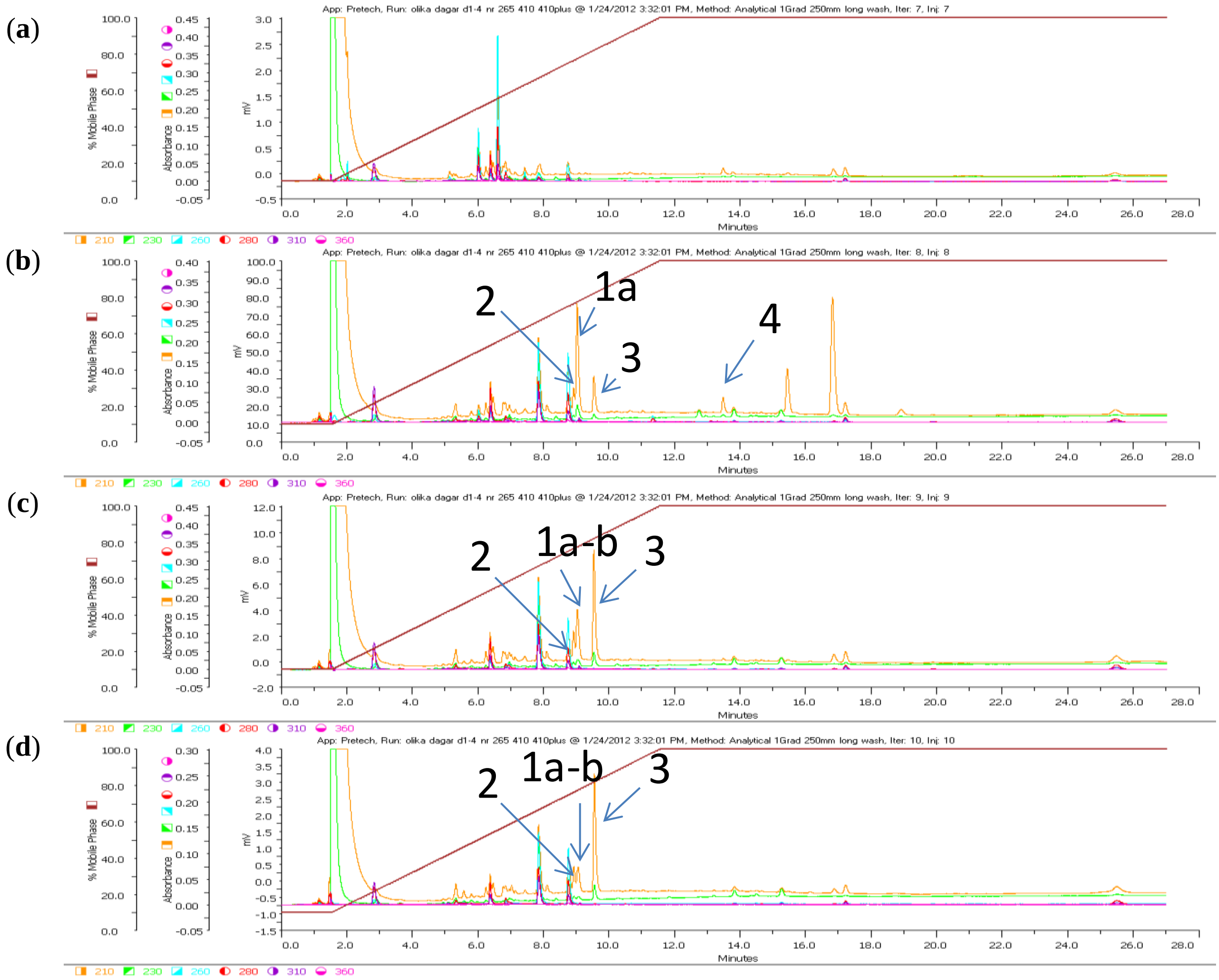

Figure S2. Ethyl acetate extract of bacterial pellet after different time of cultivation: (**a**) 44 h; (**b**) 46 h; (**c**) 52 h; (**d**) 54 h. Retention times for butenolide **1**–**4**: **1a** and **1b** = 9.04 min; **2** = 8.93 min; **3** = 9.55 min; **4** = 13.5 min.

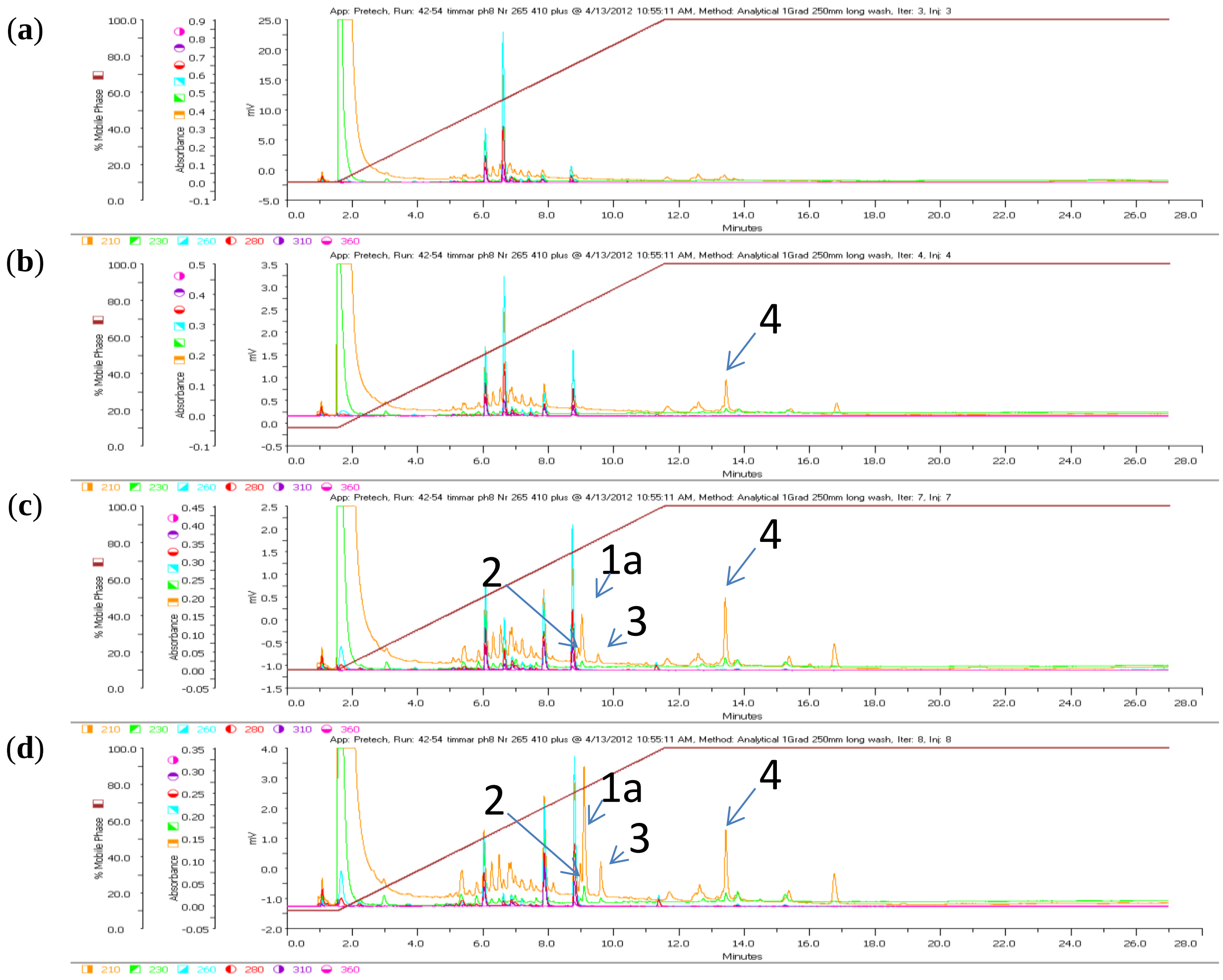

Figure S3. UV-vis spectra of butenolides **1**–**4**.

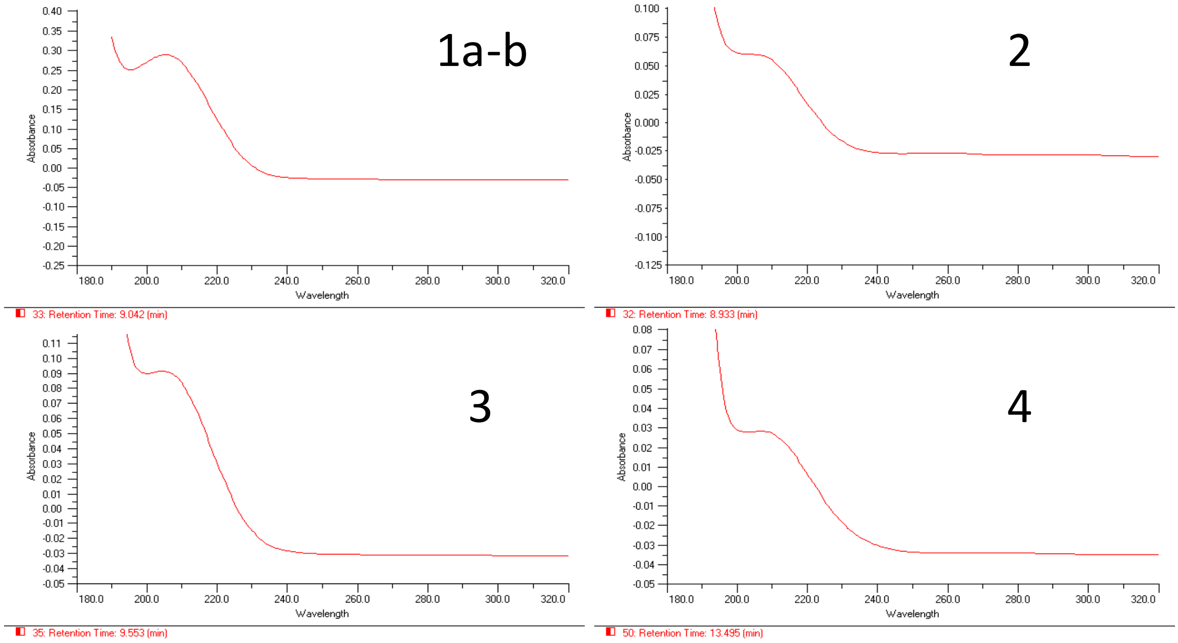

Figure S4. ^{1}H-NMR (400 MHz, $CDCl_3$) spectrum of butenolide secondary alcohol **1a**.

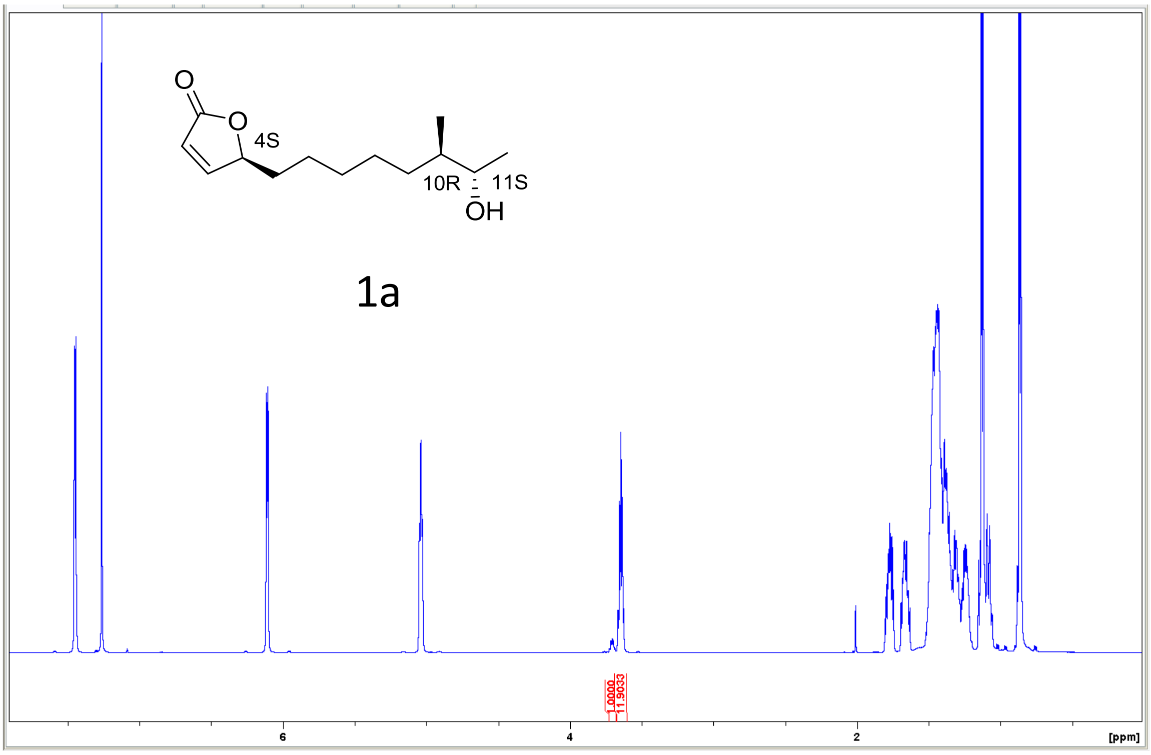

Figure S5. ^{1}H-NMR (400 MHz, $CDCl_3$) spectrum of butenolide secondary alcohols **1a** and **1b** (1:1).

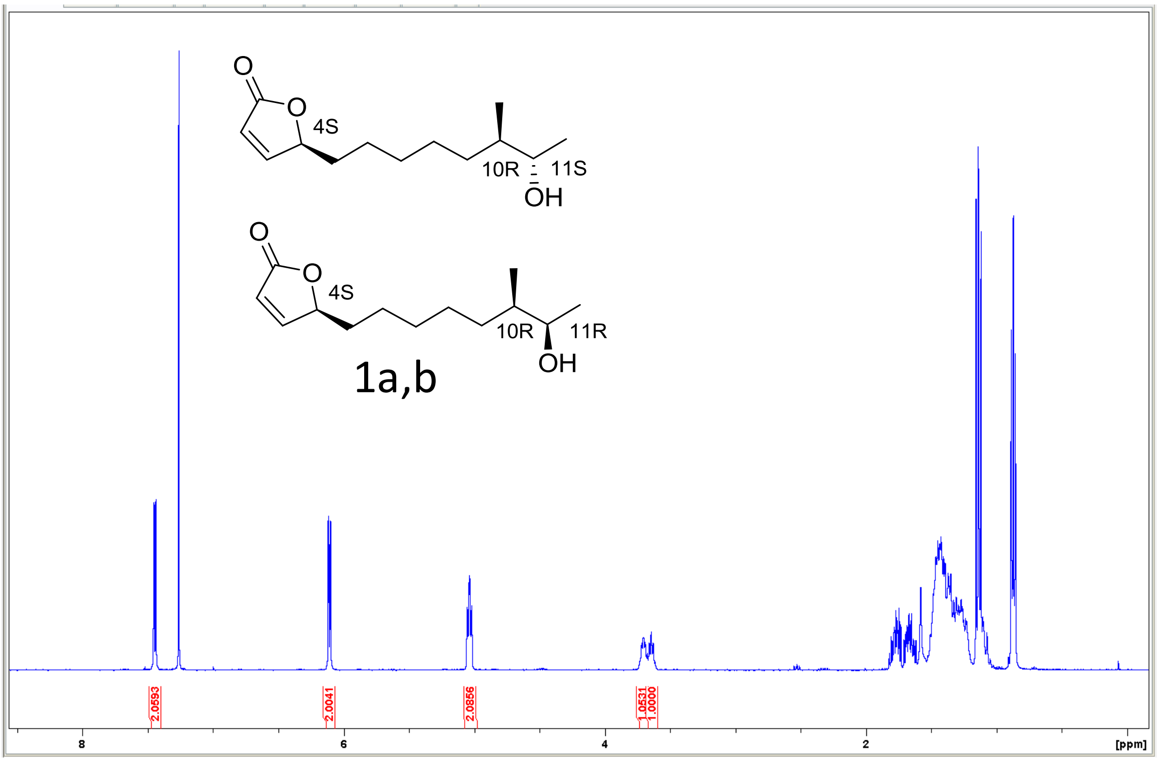

Figure S6. ^{1}H-NMR (400 MHz, $CDCl_3$) of spectrum of butenolide tertiary alcohol **2**.

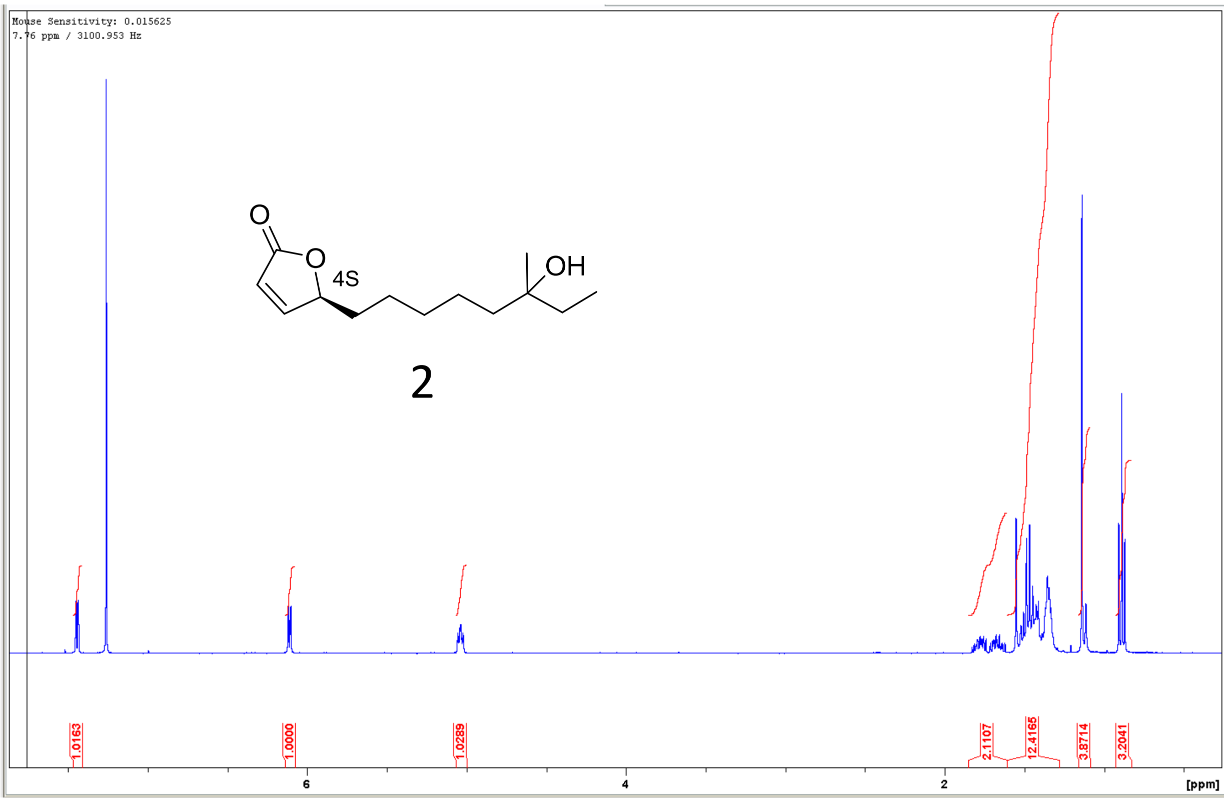

Figure S7. ^{1}H-NMR (400 MHz, $CDCl_3$) spectrum of butenolide ketone **3**.

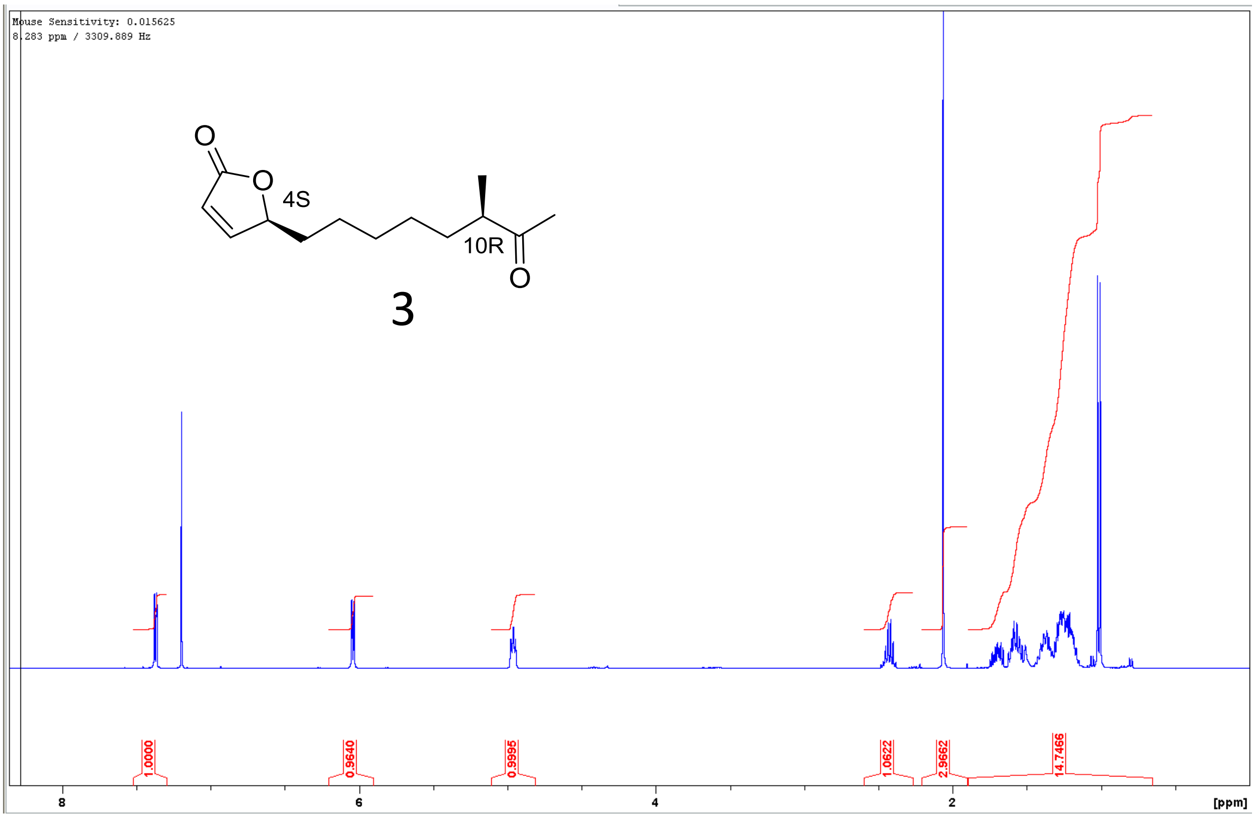

Figure S8. ^{1}H-NMR (400 MHz, $CDCl_3$) spectrum of butenolide non functional side chain **4**.

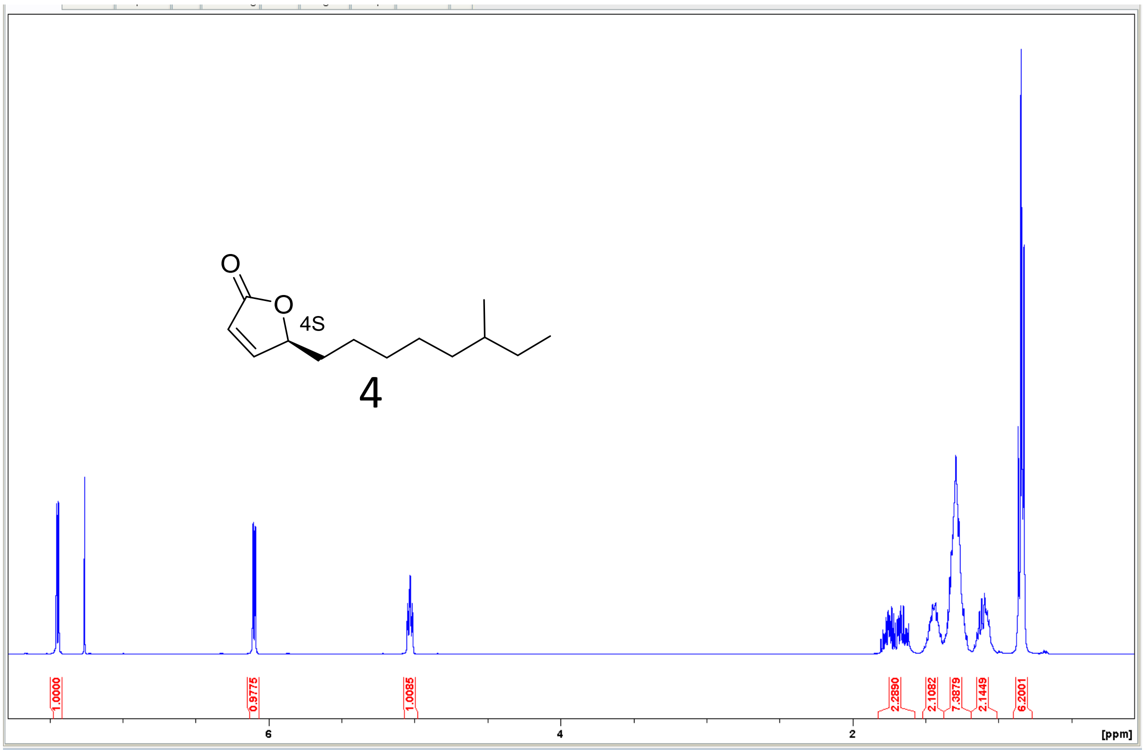

Figure S9. ^{1}H-NMR (400 MHz, $CDCl_3$) spectrum of saturated butenolide ketone **5**.

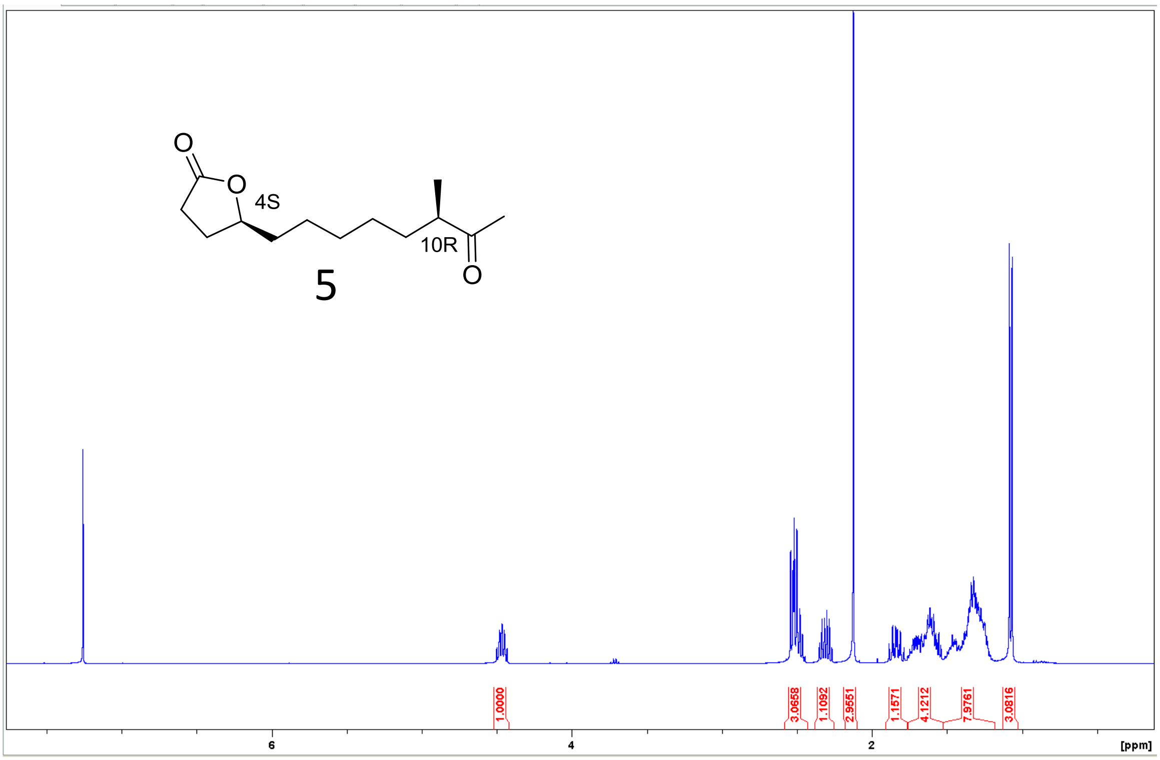

Figure S10. ^{1}H-NMR (400 MHz, $CDCl_3$) spectrum of saturated butenolide non functional side chain **6**.

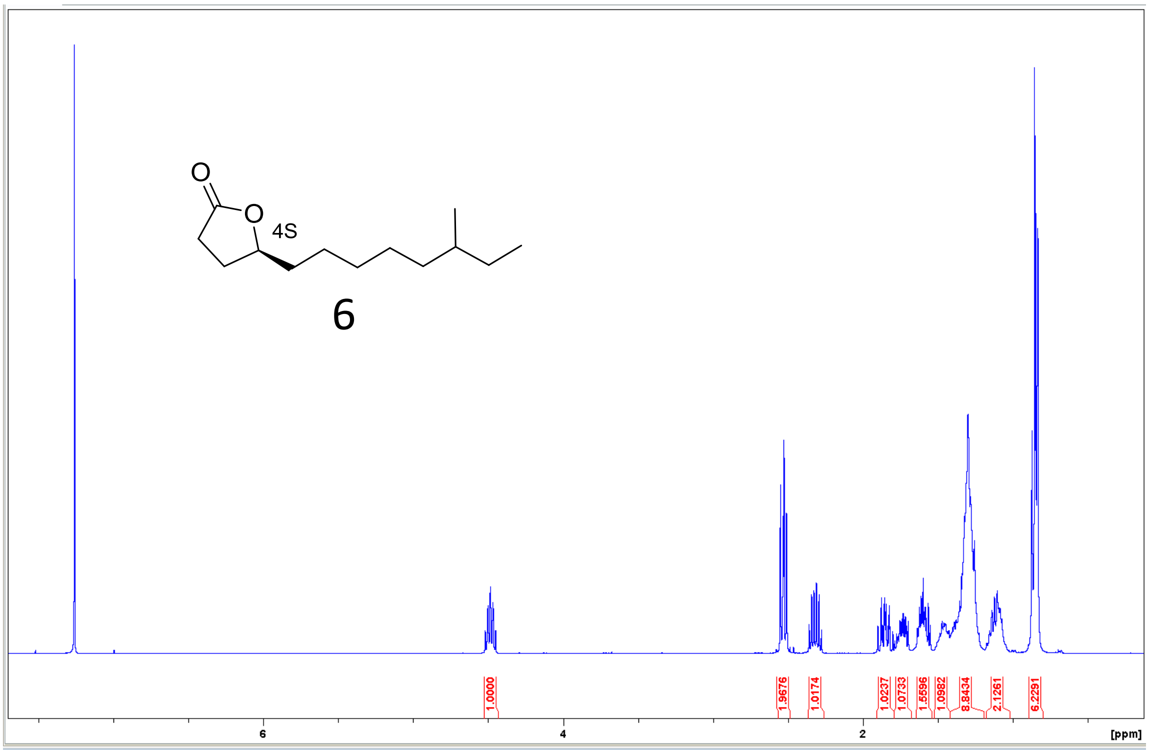

Figure S11. ^{13}C-NMR (100 MHz, $CDCl_3$) spectrum of butenolide secondary alcohol **1a**.

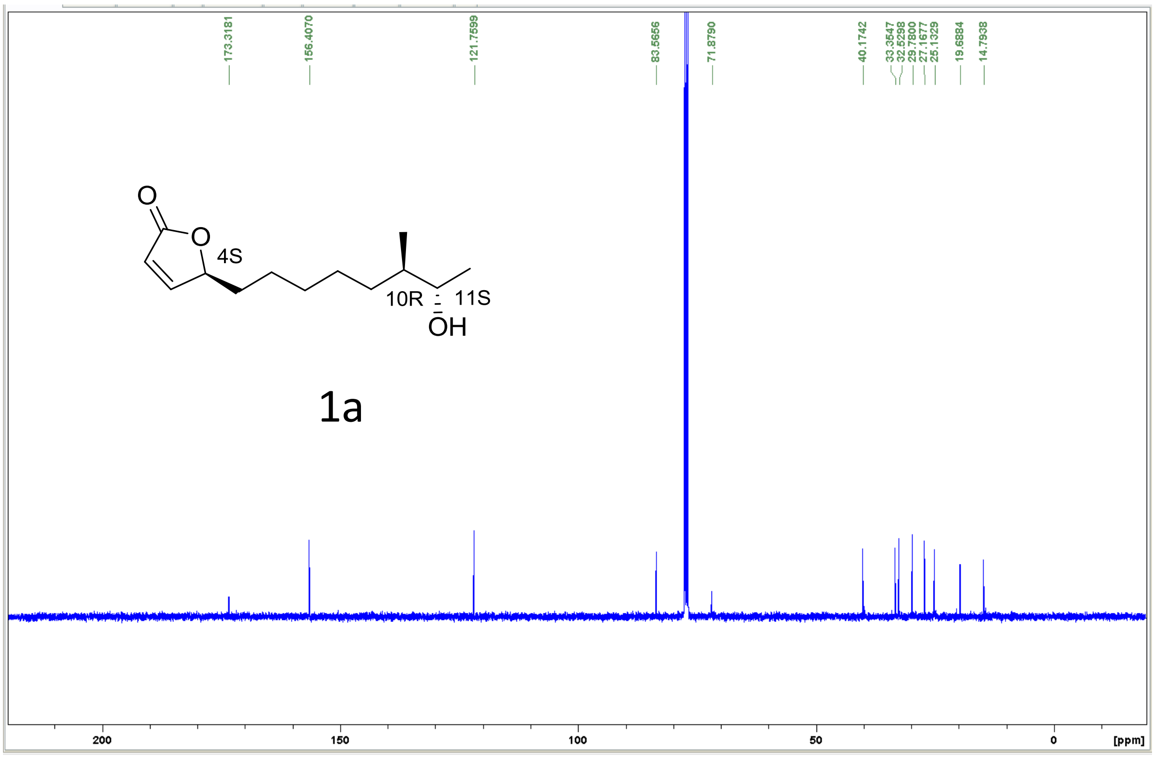

Figure S12. ^{13}C-NMR (100 MHz, $CDCl_3$) of spectrum of butenolide secondary alcohols **1a–b**.

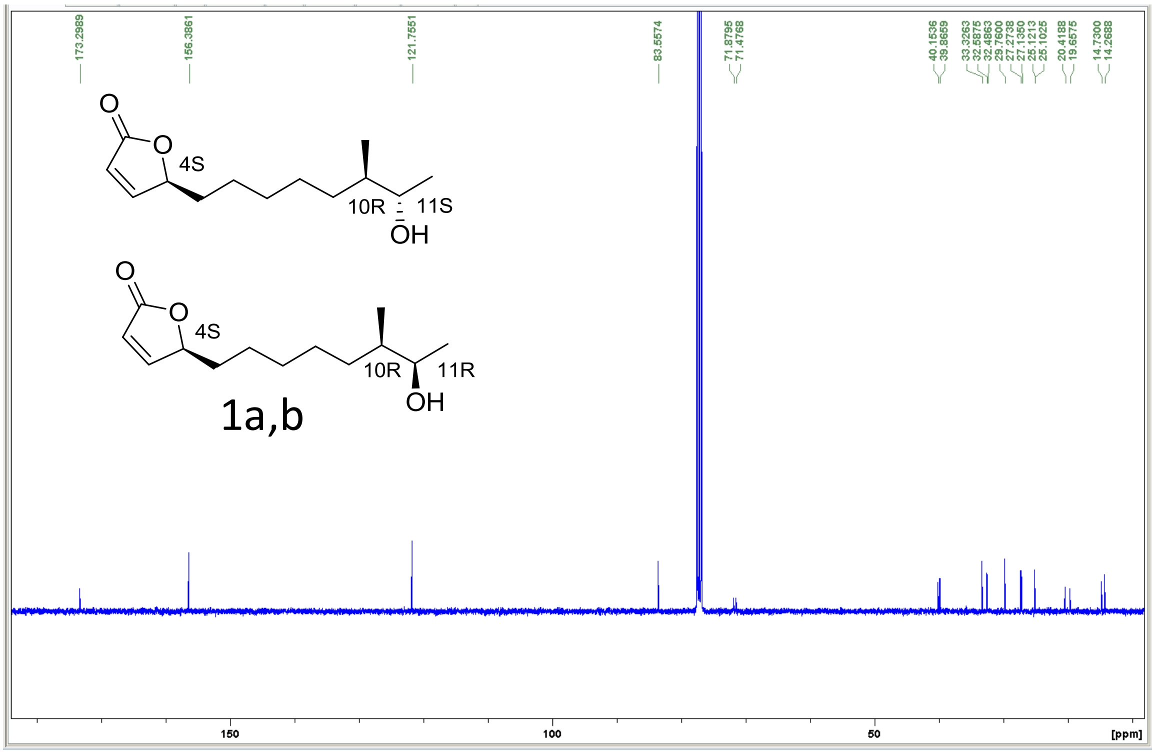

Figure S13. ^{13}C-NMR (100 MHz, $CDCl_3$) spectrum of butenolide tertiary alcohol **2**.

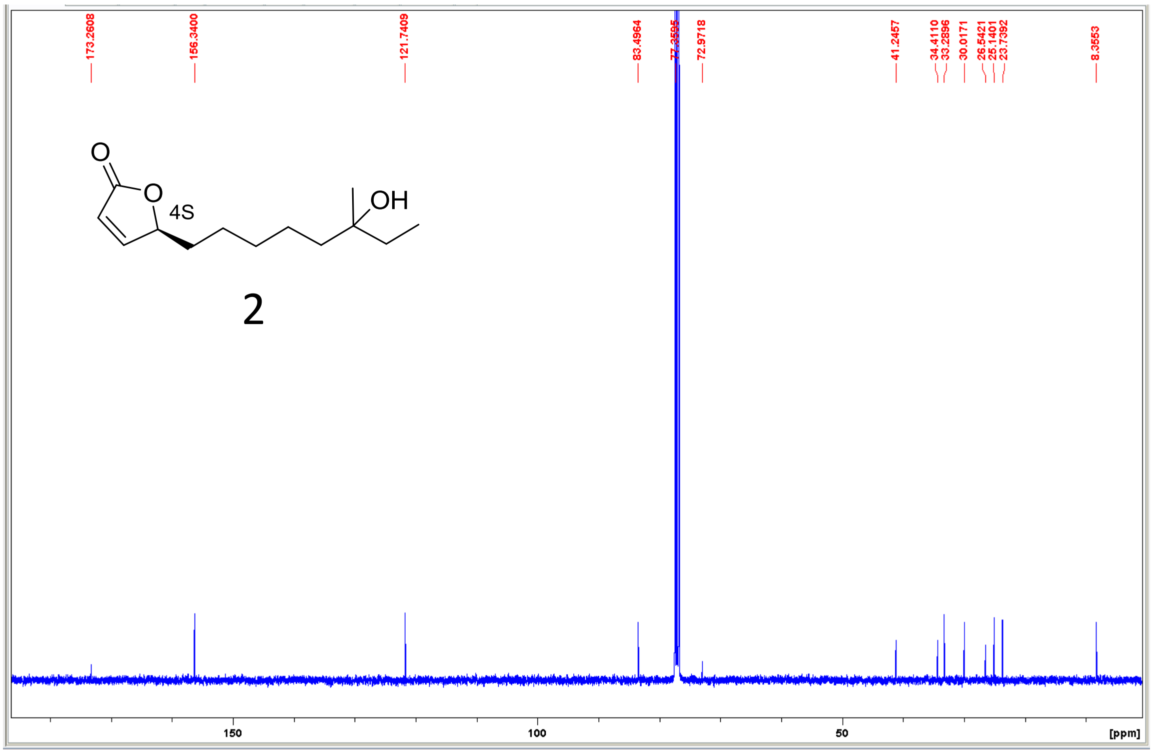

Figure S14. ^{13}C-NMR (100 MHz, $CDCl_3$) spectrum of butenolide ketone **3**.

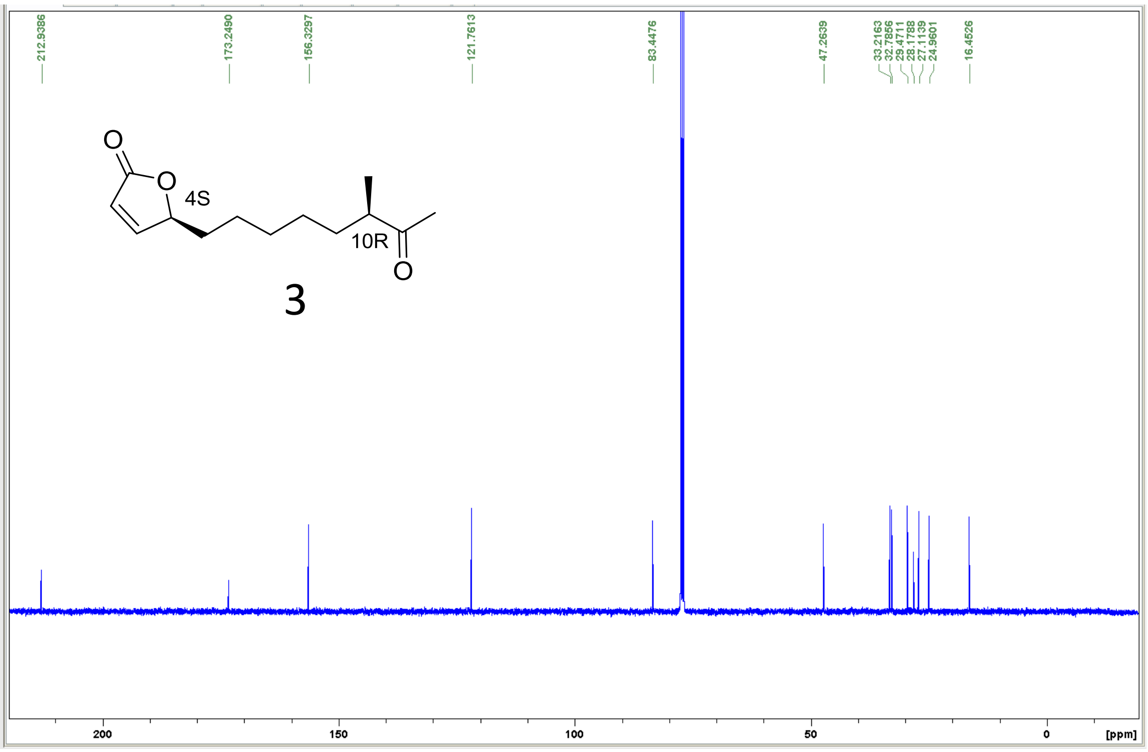

Figure S15. ^{13}C-NMR (100 MHz, $CDCl_3$) spectrum of butenolide with non functional side chain **4**.

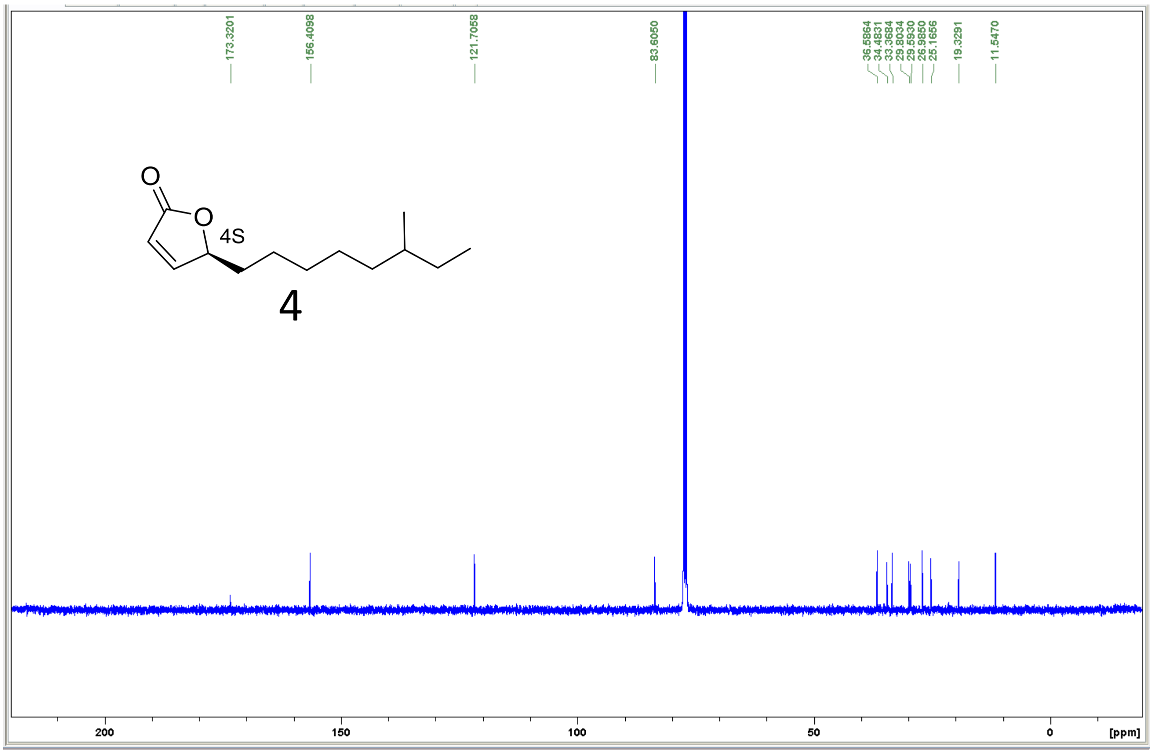

Figure S16. ^{13}C-NMR (100 MHz, $CDCl_3$) spectrum of saturated butenolide ketone **5**.

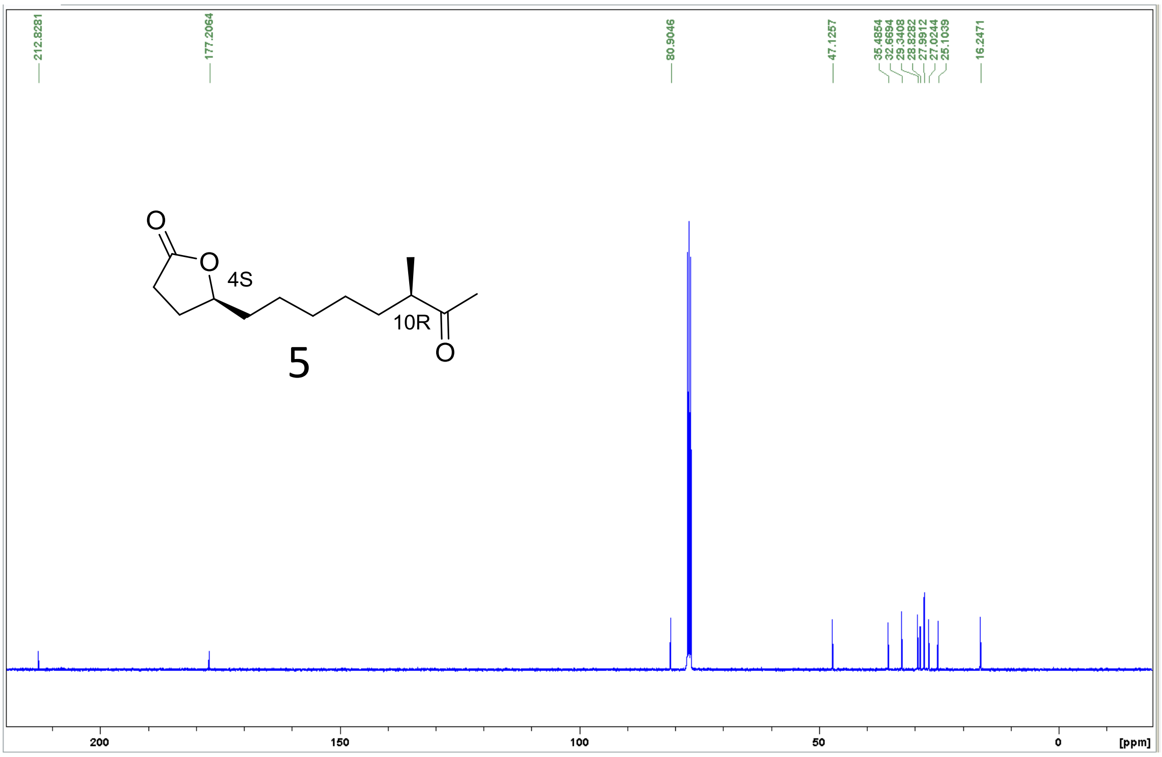

Figure S17. ^{13}C-NMR (100 MHz $CDCl_3$) spectrum of saturated butenolide **6**.

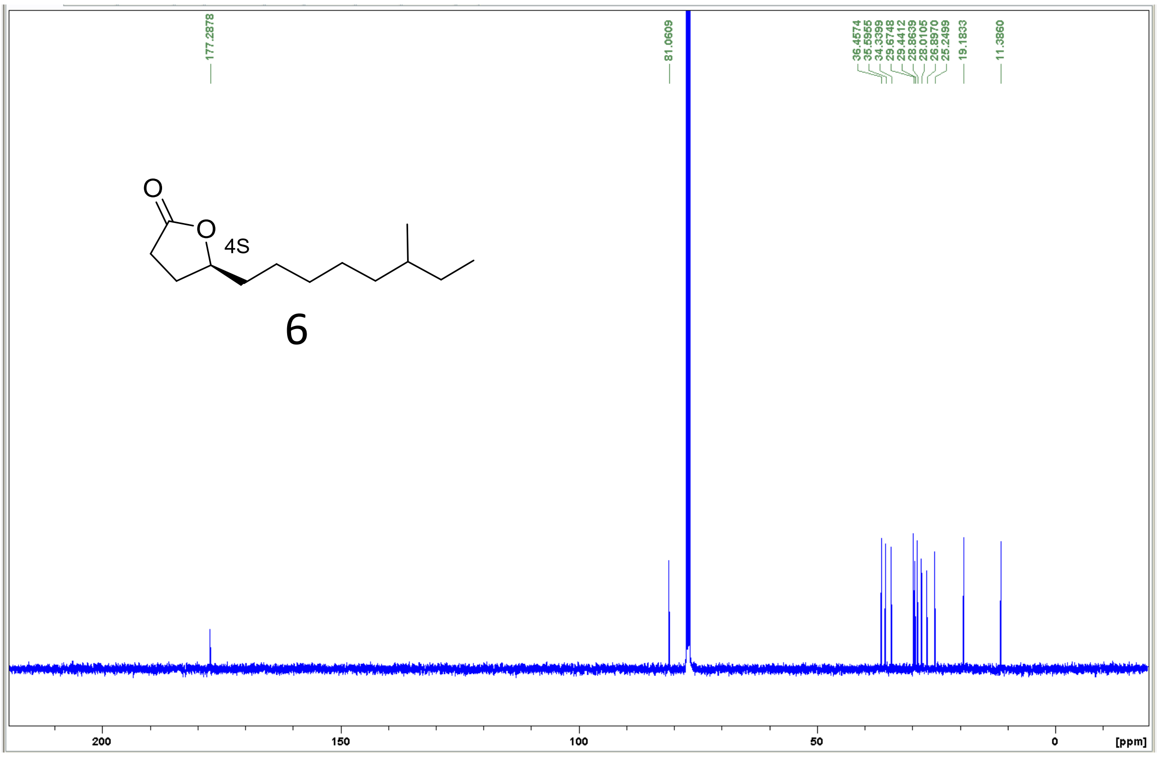

Figure S18. Phylogenetic analysis of *Streptomyces* sp. AW28M48.

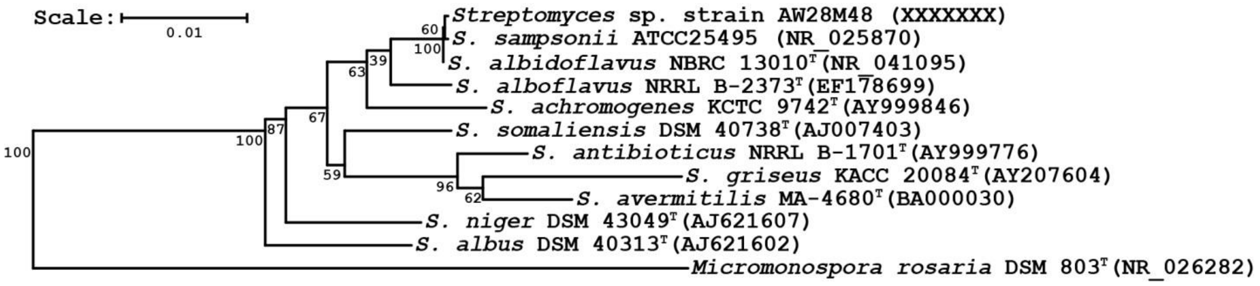

Phylogenetic relationships between *Streptomyces* sp. strain AW28M48 and selected *Streptomyces* type strains based on almost complete 16s rRNA sequences. The tree was constructed using online web-tools at the Ribosomal Database Project [1,2]. Percentages at nodes represent levels of bootstrap support from 100 resampled datasets. The bar indicates 1% estimated sequence divergence. *Micromonospora rosaria* DSM 803 was used as an outgroup. The phylogenetic tree shows that *Streptomyces*. sp. strain AW28M48 is very much related to *S. albidoflavus*. The taxonomy of *Streptomyces* belonging to this clade has been reevaluated [3] and based on different analysis it is proposed that many species (like *S. sampsonii*) are strains of *S. albidoflavus*.

References

1. Cole, J.R.; Wang, Q.; Cardenas, E.; Fish, J.; Chai, B.; Farris, R.J.; Kulam-Syed-Mohideen, A.S.; McGarrell, D.M.; Marsh, T.; Garrity, G.M.; *et al*. The Ribosomal Database Project: Improved alignments and new tools for rRNA analysis. *Nucleic Acids Res.* **2009**, *37*, D141–D145.
2. Ribosomal Database Project. Available online: http://rdp.cme.msu.edu/ (accessed on 21 January 2014).
3. Rong, X.; Guo, Y.; Huang, Y. Proposal to reclassify the *Streptomyces albidoflavus* clade on the basis of multilocus sequence analysis and DNA-DNA hybridization, and taxonomic elucidation of *Streptomyces griseus* subsp. solvifaciens. *Syst. Appl. Microbiol.* **2009**, *32*, 314–322.

© 2014 by the authors; licensee MDPI, Basel, Switzerland. This article is an open access article distributed under the terms and conditions of the Creative Commons Attribution license (http://creativecommons.org/licenses/by/3.0/).
